# LncRNA *ARGI* Contributes to Virus‐Induced Pancreatic *β* Cell Inflammation Through Transcriptional Activation of IFN‐Stimulated Genes

**DOI:** 10.1002/advs.202300063

**Published:** 2023-06-29

**Authors:** Itziar González‐Moro, Koldo Garcia‐Etxebarria, Luis Manuel Mendoza, Nora Fernández‐Jiménez, Jon Mentxaka, Ane Olazagoitia‐Garmendia, María Nicol Arroyo, Toshiaki Sawatani, Cristina Moreno‐Castro, Chiara Vinci, Anne Op de Beek, Miriam Cnop, Mariana Igoillo‐Esteve, Izortze Santin

**Affiliations:** ^1^ Department of Biochemistry and Molecular Biology University of the Basque Country Leioa 48940 Spain; ^2^ Biocruces Bizkaia Health Research Institute Barakaldo 48903 Spain; ^3^ Biodonostia Health Research Institute Gastrointestinal Genetics Group San Sebastián 20014 Spain; ^4^ Centro de Investigación Biomédica en Red de Enfermedades Hepáticas y Digestivas (CIBERehd) Barcelona 08036 Spain; ^5^ Department of Genetics Physical Anthropology and Animal Physiology University of the Basque Country Leioa 48940 Spain; ^6^ ULB Center for Diabetes Research Université Libre de Bruxelles Brussels 1070 Belgium; ^7^ Division of Endocrinology Erasmus Hospital Université Libre de Bruxelles Brussels 1070 Belgium; ^8^ Centro de Investigación Biomédica en Red de Diabetes y Enfermedades Metabólicas Asociadas (CIBERDEM) Instituto de Salud Carlos III Madrid 28029 Spain

**Keywords:** long non‐coding RNAs, pancreatic *β* cells, single nucleotide polymorphism, type 1 diabetes, viral infections

## Abstract

Type 1 diabetes (T1D) is a complex autoimmune disease that develops in genetically susceptible individuals. Most T1D‐associated single nucleotide polymorphisms (SNPs) are located in non‐coding regions of the human genome. Interestingly, SNPs in long non‐coding RNAs (lncRNAs) may result in the disruption of their secondary structure, affecting their function, and in turn, the expression of potentially pathogenic pathways. In the present work, the function of a virus‐induced T1D‐associated lncRNA named *ARGI* (Antiviral Response Gene Inducer) is characterized. Upon a viral insult, *ARGI* is upregulated in the nuclei of pancreatic *β* cells and binds to CTCF to interact with the promoter and enhancer regions of IFN*β* and interferon‐stimulated genes, promoting their transcriptional activation in an allele‐specific manner. The presence of the T1D risk allele in *ARGI* induces a change in its secondary structure. Interestingly, the T1D risk genotype induces hyperactivation of type I IFN response in pancreatic *β* cells, an expression signature that is present in the pancreas of T1D patients. These data shed light on the molecular mechanisms by which T1D‐related SNPs in lncRNAs influence pathogenesis at the pancreatic *β* cell level and opens the door for the development of therapeutic strategies based on lncRNA modulation to delay or avoid pancreatic *β* cell inflammation in T1D.

## Graphical abstract

Long non‐coding RNAs (lncRNAs) are emerging as regulators for several molecular processes that are important for human diseases. Herein, the function of a type 1 diabetes (T1D)‐associated lncRNA named *ARGI* is described. *ARGI* modulates the transcription of virus‐induced *IFNβ* and interfer on stimulated genes (ISGs) in pancreatic *β* cells in an allele‐specific manner.

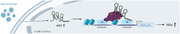

## 1 Introduction

Type 1 diabetes (T1D) is a chronic autoimmune disease characterized by the specific destruction of insulin‐producing pancreatic *β* cells that leads to impaired insulin production and increased blood glucose levels.^[^ ^1^ ^]^ During the initial stages of the disease, immune cells infiltrate pancreatic islets, generating a pro‐inflammatory environment (insulitis) that is tightly controlled by the release of soluble pro‐inflammatory mediators by both pancreatic *β* cells and immune cells.^[^ ^2^ ^]^

Over the past years, several studies have pointed to the role of T1D candidate genes in the regulation of the pro‐inflammatory process that precedes the autoimmune destruction of pancreatic *β* cells in T1D.^[^ ^3^, ^4^, ^5^, ^6^ ^]^ The molecular mechanisms by which most T1D candidate genes influence T1D pathogenesis remain to be clarified. Accumulating evidence suggests that T1D risk genes interplay with viral infections in pancreatic *β* cells, promoting an imbalanced antiviral and pro‐inflammatory response that culminates in the autoimmune destruction of insulin‐producing cells.^[^ ^3^, ^4^, ^5^, ^6^ ^]^ Indeed, the role of viral infections in T1D development is supported by clinical and epidemiological data.^[^ ^7^, ^8^, ^9^, ^10^, ^11^, ^12^ ^]^

Although inflammation‐ and virus‐induced activation of specific transcription factors (IRF7, NF*κ*B, STAT1, and STAT2, among others) is certainly a contributory factor to gene expression changes associated with *β* cell failure,^[^ ^13^, ^14^, ^15^ ^]^ recent studies have linked long non‐coding RNAs (lncRNAs) to the regulation of innate immune responses in different cell types.^[^ ^16^, ^17^, ^18^ ^]^ LncRNAs are non‐coding RNAs with a length of 200 nucleotides or more with a structure similar to the one of protein‐coding genes.^[^ ^19^ ^]^ Several studies have implicated lncRNAs in biological and cellular processes related to inflammation, including activation of the innate antiviral immune response through the activation of pattern recognition receptor (PRR)‐related signal transduction, regulation of innate immune‐associated chemokines, and other inflammatory genes.^[^ ^20^, ^21^, ^22^ ^]^ The function of non‐coding RNAs is only beginning to emerge but there is already strong evidence of their involvement in disease, including inflammatory and autoimmune disorders.^[^ ^5^, ^23^, ^24^, ^25^ ^]^

Genome‐wide association studies (GWAS) have identified a large number of single nucleotide polymorphisms (SNPs) predisposing to immune diseases.^[^ ^26^ ^]^ Only a small fraction of these SNPs is located within protein‐coding genes and the majority map to non‐coding regions of the human genome.^[^ ^27^ ^]^ Around 10% of the SNPs associated to immune disorders lie in lncRNAs, suggesting that risk variants in these non‐coding molecules could alter their function and deregulate gene expression networks potentially important for pancreatic *β* cell function and T1D development.^[^ ^28^ ^]^

A combination of expression data and DNA sequence variation in T1D previously led to the description of an antiviral gene expression network linked to T1D susceptibility named IDIN (IRF7‐driven inflammatory network).^[^ ^29^ ^]^ In this study, Heinig et al showed that the genotype of an intergenic T1D risk SNP (rs9585056) correlated with the expression of the IDIN network in immune cells. In the current study, we show that rs9585056 is not intergenic but is located in NONHSAT233405.1 or *ARGI* (Antiviral Response Gene Inducer), a lncRNA gene that is upregulated by Coxsackievirus infections in pancreatic *β* cells.

We functionally characterized *ARGI* and demonstrated that it regulates the expression of antiviral and pro‐inflammatory genes in pancreatic *β* cells. *ARGI* participates in pancreatic *β* cell inflammation via transcriptional regulation of interferon‐stimulated genes (ISGs). Since type I IFN signaling plays a crucial role in T1D‐related *β* cell dysfunction, *ARGI* may be functionally implicated in the pathogenesis of the disease.^[^ ^2^ ^]^ These results serve as a proof of concept of the potential implication of T1D‐associated lncRNAs in the dysfunction of pancreatic *β* cells in T1D, and open a new avenue for the development of therapeutic approaches based on lncRNA expression modification.

## 2 Results

### 2.1 *ARGI* Is a Nuclear LncRNA Upregulated in EndoC‐*β*H1 Cells Upon a Viral Infection

To identify potential lncRNAs associated with T1D, the genomic locations of all T1D‐associated SNPs (GWAS catalog; https://www.ebi.ac.uk/gwas) were intersected with the genomic location of all lncRNAs annotated in NONCODE version 6 (http://www.noncode.org/index.php). Through this approach, we identified 69 lncRNAs harboring at least one SNP associated with the disease (Figure S1, Supporting Information). Of those 69, 19 lncRNAs have a T1D‐associated SNP in their exonic region. Because SNPs in lncRNAs may affect their function through the disruption of their secondary structure,^[^ ^5^, ^25^ ^]^ we assessed whether these19 SNPs alter lncRNA secondary structure. In silico predictions using RNAsnp software (Center for non‐coding RNA in Technology and Health) revealed that the secondary structure of 10 lncRNAs was altered by at least one of the T1D‐associated SNPs.

**Figure 1:**
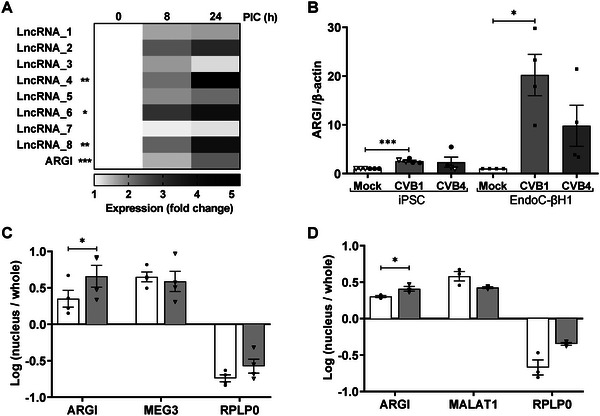
*ARGI* is a nuclear lncRNA upregulated in pancreatic *β* cells upon viral infection. A) EndoC‐*β*H1 cells were exposed to intracellular PIC (0.25 µg L^−1^) for 8 or 24 h and the expression of ten T1D‐associated lncRNAs was determined by qPCR. One of ten lncRNAs was not detected and the expression of the other nine lncRNAs in EndoC‐*β*H1 cells is presented as a heatmap (fold change versus PIC 0 h). Results are means of three independent experiments; *p < 0.05, **p < 0.01, ***p < 0.001 for 24 h versus 0 h; Student's *t*‐test. B) Induced pluripotent stem cell (iPSC)‐derived pancreatic islet‐like aggregates and EndoC‐*β*H1 were left uninfected (Mock) or infected with CVB1 or CVB4 for 24 h. *ARGI* expression was assessed by qPCR and normalized to the reference gene *β*‐actin. Data are means ± SEM of three independent HEL115.6 iPSC differentiations (white triangles), three independent 1023A iPSC differentiations (black circles), and four independent EndoC‐*β*H1 samples (black squares); ***p < 0.001 and *p < 0.05; Student's *t*‐test. C) EndoC‐*β*H1 cells were left untransfected (white bars) or exposed to PIC (0.25 µg L^−1^) for 24 h (grey bars). Relative *ARGI* expression was determined in nucleus and whole cell extracts, using *MEG3* and *RPLP0* as controls for the respective fractions. Amounts of specific nuclear RNA were measured by qPCR and compared to the total amount of RNA in the whole cell extract. Data are expressed as a logarithm (nucleus/whole) and are means±SEM of 4 independent experiments; *p < 0.05; Student's *t*‐test. D) EndoC‐*β*H1 cells were left uninfected (white bars) or infected with CVB1 (MOI 0.05, grey bars). Relative *ARGI* expression was determined in nucleus and whole cell extracts, using *MALAT* and *RPLP0* as controls for nuclear and whole cell fractions, respectively. Amounts of specific nuclear RNA were measured by qPCR and compared to the total amount of RNA in the whole cell. Data are expressed as a logarithm (nucleus/whole) and are means±SEM of 3 independent experiments; *p < 0.05; Student's *t*‐test.

We next examined whether these 10 T1D‐associated lncRNAs were expressed in pancreatic *β* cells and modulated by viral infections. To this end, we analyzed their expression in the human *β* cell line EndoC‐*β*H1 in basal condition and after transfection with polyinosinic‐polycytidylic acid (PIC), a molecule that simulates viral dsRNA produced during viral infections. Nine out of the analyzed 10 lncRNAs were expressed in pancreatic *β* cells, and as shown in **Figure** **1**A, all except *LncRNA_7* were upregulated 8 and/or 24 h after PIC transfection.

One of the PIC‐upregulated T1D‐associated lncRNAs, *argi* (Antiviral Response Gene Inducer), harbors an SNP (rs9585056) in its third exon that was previously described as an intergenic SNP that acts as an eQTL regulating expression of the IDIN antiviral gene network in monocytes and macrophages.^[^ ^29^ ^]^ Our mapping of T1D‐associated SNPs against lncRNAs showed, however, that this SNP is not intergenic but falls into the hitherto uncharacterized lncRNA *argi*. Moreover, our in silico prediction suggested that the SNP disrupts *ARGI*’s secondary structure by affecting one of its loops (Figure S2, Supporting Information). Considering that enteroviral infections such as coxsackievirus B (CVB) may contribute to T1D pathogenesis by infecting pancreatic *β* cells,^[^ ^12^, ^30^, ^31^, ^32^ ^]^ we set out to characterize the function of *ARGI* at the pancreatic *β* cell level.

LncRNA *ARGI* is ubiquitously expressed in human tissues at different expression levels (Figure S3A, Supporting Information). The highest expression is observed in the EndoC‐*β*H1 cell line, followed by liver, colon, thyroid, and placenta. Brain and thymus had the lowest expression of *ARGI*. In human pancreatic islets, its expression level is relatively low, and interestingly, it is similar in human primary pancreatic *α* and *β* cells (Figure S3B, Supporting Information).

We next examined whether CVB infections modify *ARGI* expression in pancreatic *β* cells. To this end, we differentiated human induced pluripotent stem cells (iPSCs) into pancreatic islet‐like aggregates using a seven‐stage method that mimics embryonic *β* cell development. At the end of the differentiation, the iPSC‐derived aggregates contained 47.9% ± 3.48% *β* cells, 2.7% ± 1.22% *α* cells, and 0.7% ± 0.08% *δ* cells. We infected these human iPSC‐derived islet‐like aggregates with two diabetogenic CVB serotypes, namely CVB1 and CVB4. CVB1 increased *argi* expression, while the induction with CVB4 was more variable (Figure 1B). Similar results were observed when EndoC‐*β*H1 cells were infected with CVB1 or CVB4 (Figure 1B). Infections of EndoC‐*β*H1 cells with either serotype increased *ARGI* expression, although only CVB1 infection reached significance.

Considering that cellular location often determines lncRNA function,^[^ ^19^, ^33^, ^34^, ^35^ ^]^ we next examined the subcellular localization of *ARGI* in the human *β* cell line EndoC‐*β*H1. In basal condition, *argi* was preferentially expressed in the nucleus of EndoC‐*β*H1 cells (Figure 1C), and its nuclear expression was upregulated after 24h PIC exposure. In keeping with the results of this viral dsRNA mimic, CVB1 infection induced nuclear *ARGI* expression in EndoC‐*β*H1 cells (Figure 1D).

### 2.2 *ARGI* Upregulation in Pancreatic *β* Cells Leads to Hyperactivation of An Inflammatory and Antiviral Gene Signature

**Figure 2:**
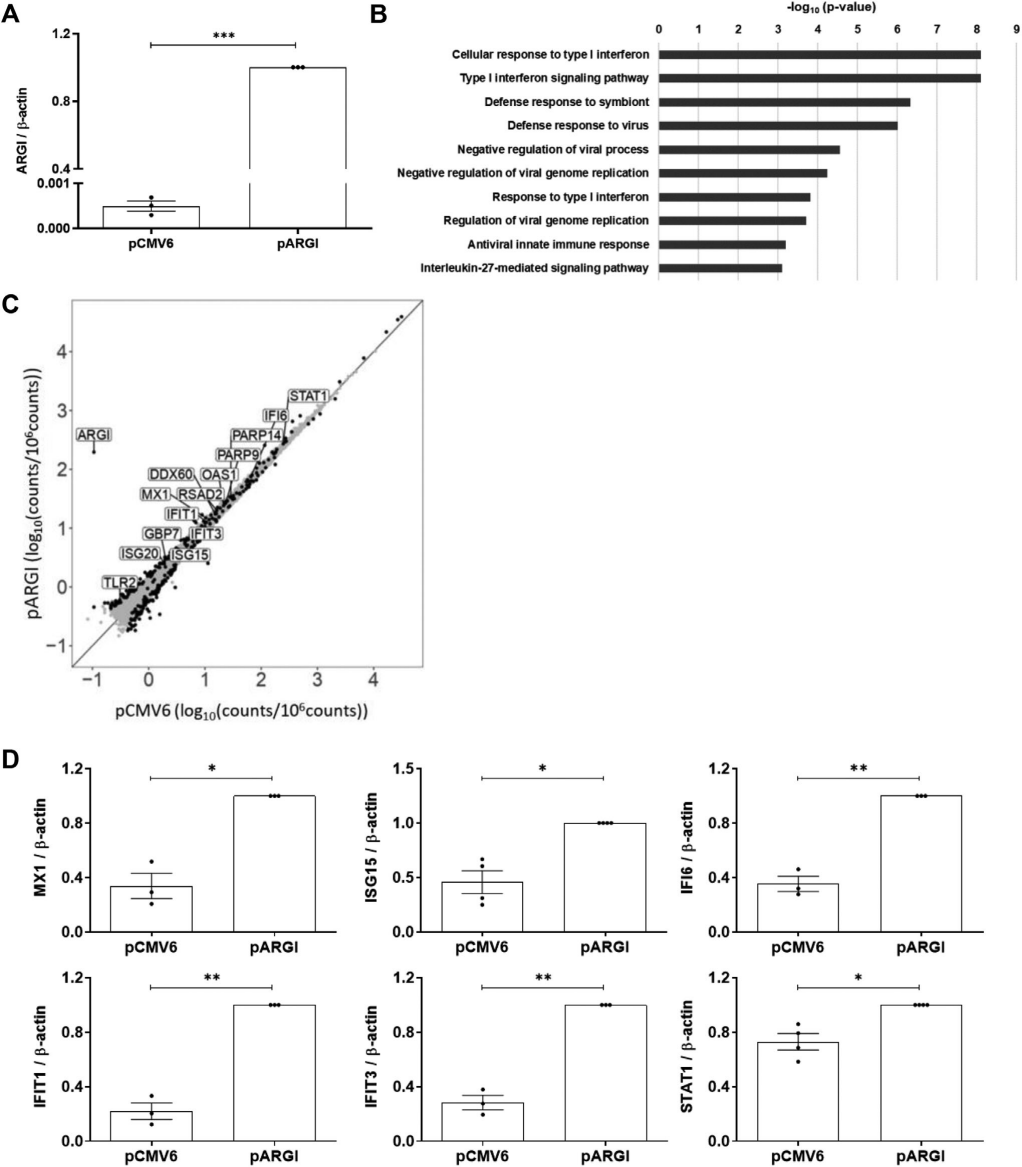
*ARGI* upregulation in pancreatic *β* cells induces an inflammatory and antiviral gene signature. A) EndoC‐*β*H1 cells were transfected with an empty overexpression plasmid (pCMV6) or with a plasmid overexpressing *ARGI* (pARGI). *ARGI* expression was determined by qPCR and normalized to the reference gene *β*‐actin. Results are means±SEM of 3 independent experiments; ***p < 0.001; Student's *t*‐test. B) Gene Ontology analysis showed enrichment of inflammatory and antiviral pathways in *ARGI*‐overexpressing pancreatic *β* cells. C) Scatterplot showing differentially expressed genes (black dots) in *ARGI*‐overexpressing EndoC‐*β*H1 cells (pARGI) compared to pCMV6‐transfected control cells. Expression values are presented as Log_10_ (counts/10^6^ counts). *ARGI* and genes from the IDIN network are indicated. D) EndoC‐*β*H1 cells were transfected with a control empty plasmid (pCMV6) or with an *ARGI*‐overexpressing plasmid (pARGI). Expression of *MX1*, *ISG15*, *IFI6*, *IFIT1*, *IFIT3*, and *STAT1* genes was determined by qPCR and normalized to the reference gene *β*‐actin. Results are means ± SEM of three independent experiments; **p < 0.01 and *p < 0.05; Student's *t*‐test.

In order to dissect the biological impact of *argi* upregulation in response to viral dsRNA (PIC), we performed RNA‐sequencing of *argi*‐overexpressing pancreatic *β* cells. *argi* was upregulated in the EndoC‐*β*H1 cell line using an overexpression vector (**Figure** **2**A). Interestingly, Gene Ontology (GO) enrichment analysis revealed that the upregulated genes were enriched in pathways related to antiviral responses and type I IFN signaling (cellular response to type I IFNs, type I IFN signaling pathway, defense response to virus, among others) (Figure 2B).

Among the upregulated genes, members of the IDIN gene expression network were significantly overrepresented (Figure 2C). Among the 17,249 transcripts detected by RNA‐seq, 430 (2.5%) were genes of the IDIN network, and 4.3% of the significantly upregulated genes were IDIN members (*p*‐value: 0.0188). The top‐upregulated IDIN genes included the following ISGs: *MX1, ISG15, IFI6, IFIT1, IFIT3*, and *STAT1*. Expression of these top six upregulated genes in *ARGI*‐overexpressing *β* cells was confirmed in an independent sample set by qPCR (Figure 2D).

Considering that *ARGI* is implicated in pancreatic *β* cell inflammation driven by viral infections, we next analyzed whether it is involved in virus‐induced *β* cell apoptosis. To this end, we determined PIC‐induced caspase‐3/7 activation in *ARGI*‐overexpressing or *ARGI*‐silenced EndoC‐*β*H1 cells (Figure S4A,B, Supporting information). As shown in Figure S4C,D (Supporting Information), intracellular PIC induced caspase‐3/7 activation, but neither up‐ or downregulation of *ARGI* affected PIC‐induced EndoC‐*β*H1 cell apoptosis.

### 2.3 *ARGI* Participates in The Regulation of Virus‐Induced IFN‐*β* and ISG Expression in Pancreatic *β* Cells

**Figure 3:**
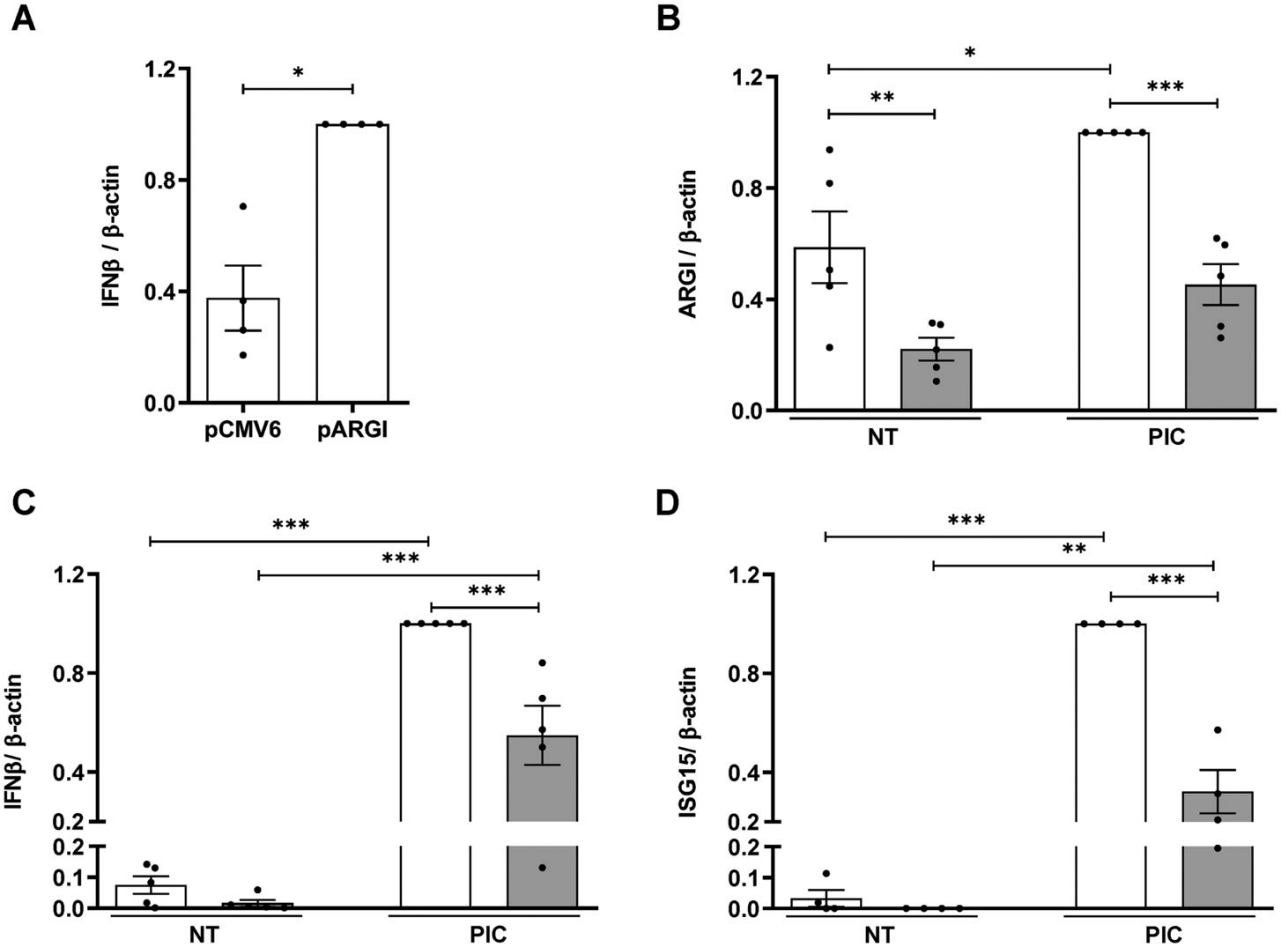
*ARGI* participates in the regulation of virus‐induced *IFNβ* and ISG expression in pancreatic *β* cells. A) EndoC‐*β*H1 cells were transfected with pCMV6 or pARGI. *IFNβ* expression was determined by qPCR and normalized to the reference *β*‐actin. Results are means ± SEM of four independent experiments. *p < 0.05; Student's *t*‐test. B) Transcriptional activation of *ARGI* was inhibited using CRISPRi. EndoC‐*β*H1 cells were transfected with an empty CRISPRi vector (white bars) or with a CRISPRi vector harboring a sgRNA targeting a conserved NF*κ*B binding site in *ARGI*’s regulatory region (grey bars). After 36 h of transfection, cells were left non‐transfected (NT) or transfected with PIC (0.25 µg L^−1^) for 24 h. Expression of *ARGI* (B), *IFNβ* (C), and *ISG15* (D) was determined by qPCR and normalized to the reference gene *β*‐actin. Results are means ± SEM of 4–5 independent experiments; ***p < 0.001, **p < 0.01, and *p < 0.05 as indicated; Student's *t*‐test or ANOVA followed by Bonferroni's multiple comparisons test.

Because most of the *ARGI*‐upregulated IDIN genes were ISGs,^[^ ^36^ ^]^ we asked whether *IFNβ* expression was induced in *argi*‐overexpressing *β* cells. *argi* overexpression increased *IFNβ* expression by 60% in pancreatic *β* cells (**Figure** **3**A), suggesting that *argi* might, at least in part, regulate ISGs through *IFNβ* induction.

To test whether *ARGI* is implicated in IFN*β* transcription, we deleted *ARGI* by CRISPR‐Cas9 in pancreatic *β* cells (Figure S5A, Supporting Information). Due to the low replication rate of EndoC‐*β*H1 cells, we could not select a complete knockout clone. In basal condition, *ARGI* expression was similar in CRISPR‐Cas9‐transfected and control cells. In cells exposed to intracellular PIC, however, *ARGI* expression was 30–50% lower in CRISPR‐Cas9‐transfected cells (Figure S5B, Supporting Information). This led to a 60–70% decrease in PIC‐induced *IFNβ* and *ISG15* expression in pancreatic *β* cells (Figure S5C,D, Supporting Information).

To confirm these results, we inhibited endogenous *ARGI* expression by CRISPRi technique using a sgRNA targeting a conserved NF*κ*B binding site in a potential regulatory region close to the *argi* transcription‐starting site (Figure S6A, Supporting Information). We first confirmed that *ARGI* was partially regulated by NF*κ*B using the specific chemical inhibitor BAY 11–7082. As shown in Figure S6B, PIC‐induced *ARGI* upregulation was counteracted by NF*κ*B inhibition. Using the CRISPRi‐sgRNA, *ARGI* expression was downregulated by ≈50% both in basal and in PIC‐transfected conditions (Figure 3B). In keeping with the CRISPR‐Cas9 experiment, PIC‐induced *IFNβ* (Figure 3C) and *ISG15* (Figure 3D) expression was also reduced upon *ARGI* inhibition by 40 and 60%, respectively. These results show that *ARGI* regulates expression of *IFNβ* as well as other ISGs, such as *ISG15*.

### 2.4 *ARGI* Associates with the Transcription Factor CTCF to Bind to The Regulatory Regions of *IFNβ* and *ISG15* Genes in PIC‐Transfected Cells

**Figure 4:**
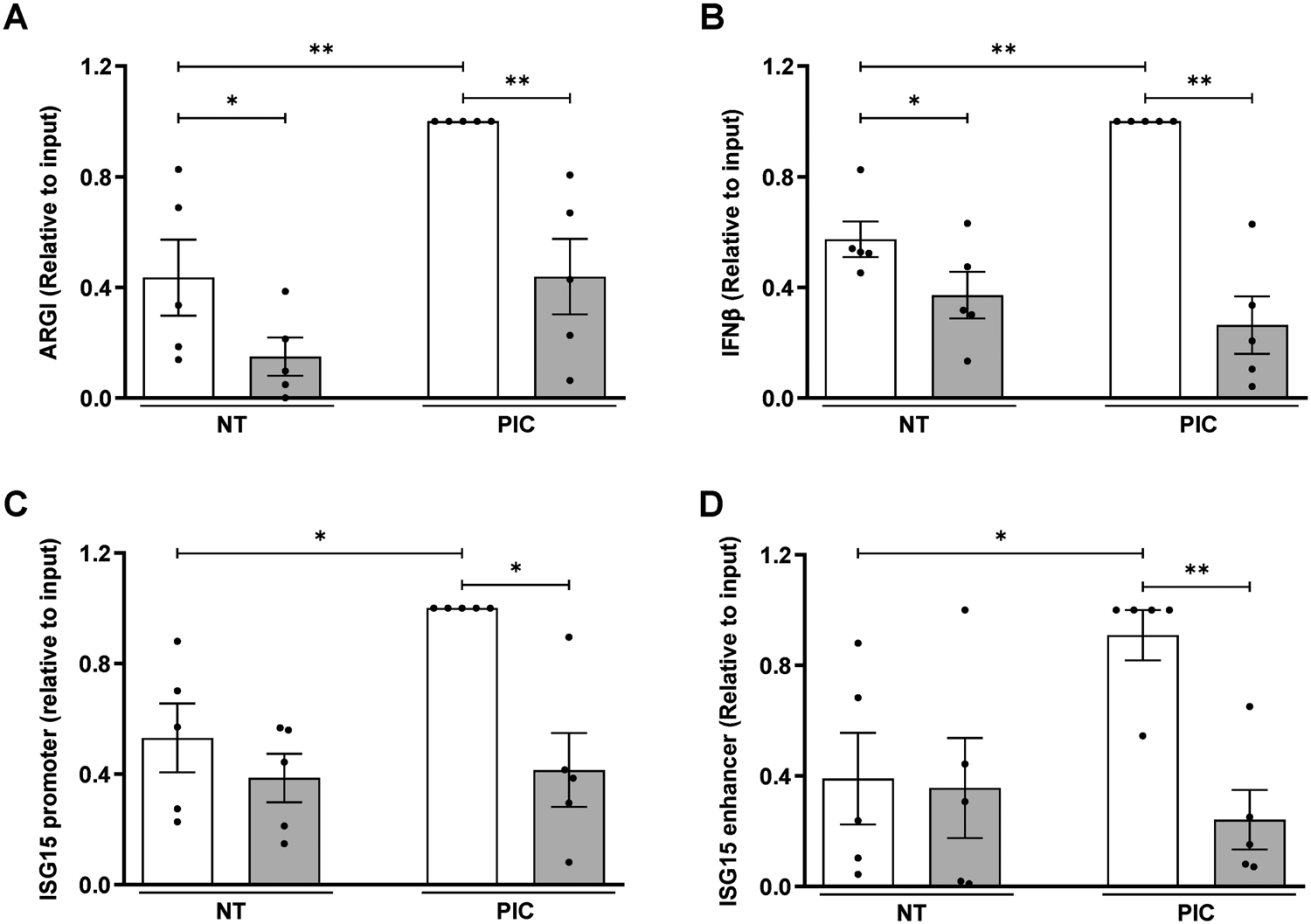
*ARGI* binds to *IFNβ* and *ISG15* regulatory regions upon viral insult. RNA antisense purification of *ARGI* was performed in non‐transfected (NT) or PIC‐transfected EndoC‐*β*H1 cells (PIC). A) *ARGI* was purified using biotinylated antisense complementary oligonucleotides (white bars); antisense oligonucleotides complementary to an unrelated similar length lncRNA were used as negative control (grey bars). B–D) *ARGI*‐bound *IFNβ* promoter (B), *ISG15* promoter (C), and *ISG15* enhancer (D) amounts were determined by qPCR. Results are expressed as relative to input and are means ± SEM of five independent experiments. **p < 0.01 and *p < 0.05 as indicated; ANOVA followed by Bonferroni's multiple comparisons test.

In order to determine the molecular mechanisms by which *argi* regulates expression of *IFNβ* and ISGs, we next performed an RNA antisense purification experiment to purify *argi*‐bound chromatin in non‐treated and PIC‐treated pancreatic *β* cells, and analyzed whether the regulatory regions of *IFNβ* and *ISG15* were present by qPCR. *argi* was purified using biotinylated antisense complementary oligonucleotides. Antisense oligonucleotides complementary to an unrelated lncRNA of similar size were used as negative control (**Figure** **4**A and Figure S7A, Supporting Information). *ARGI* purification was more efficient in PIC‐transfected cells than in non‐transfected cells, most probably due to *ARGI* induction by PIC (Figure 4A). The binding of *ARGI* to *IFNβ* and *ISG15* regulatory regions was determined by qPCR using primer pairs targeting promoter and enhancer regions of *IFNβ* and *ISG15* genes (Figure S7B,C, Supporting Information). As shown in Figure 4B–D, in basal condition, *ARGI* bound *IFNβ* promoter but it did not bind the *ISG15* promoter or *ISG15* enhancer. In PIC‐treated *β* cells, *IFNβ* promoter, *ISG15* promoter, and *ISG15* enhancer levels were increased both, compared with non‐PIC‐transfected *β* cells and relative to PIC‐transfected cells transfected with an unrelated control lncRNA.

**Figure 5:**
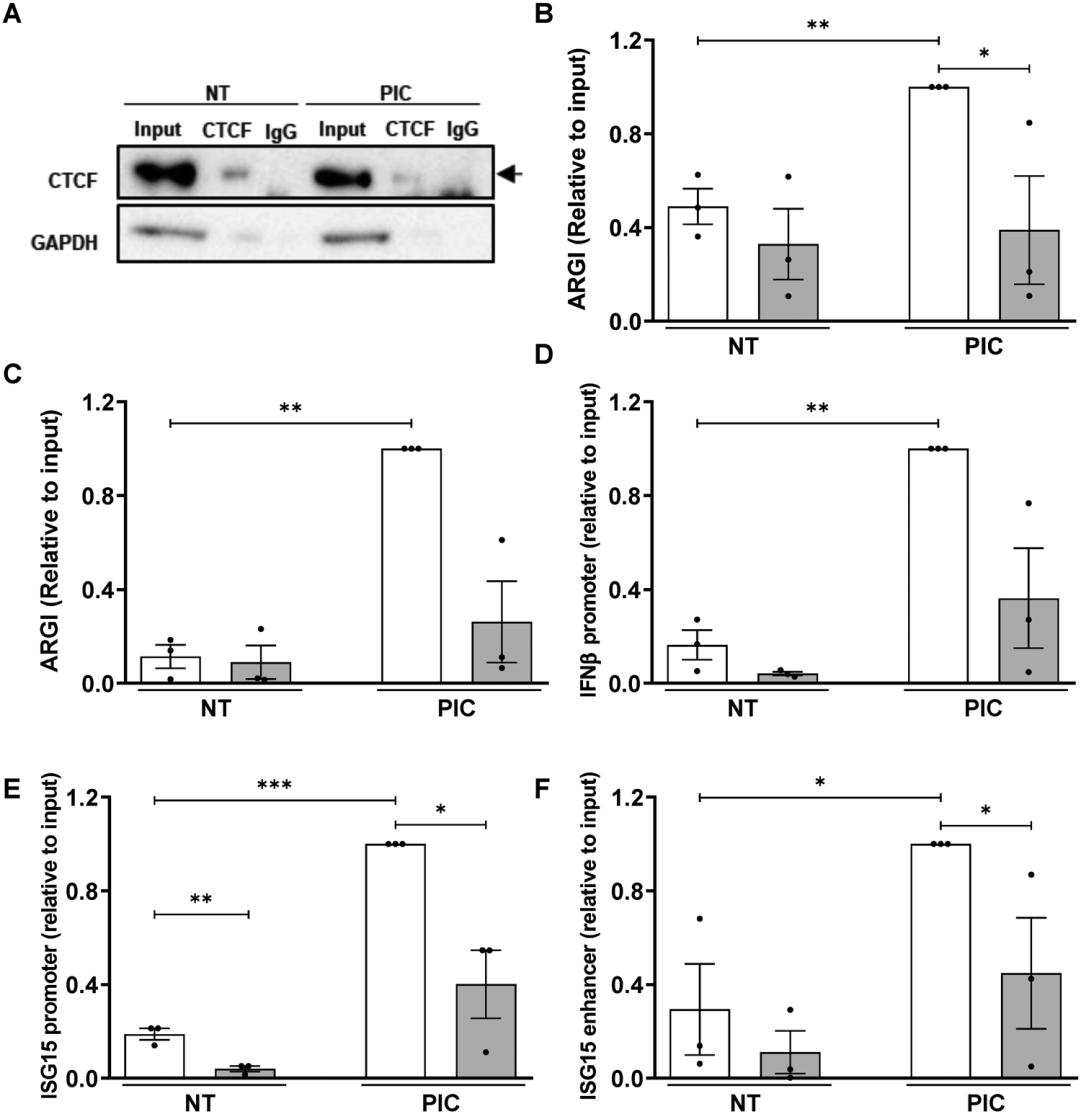
*ARGI* associates with the transcription factor CTCF to bind the regulatory regions of *IFNβ* and *ISG15* genes in viral dsRNA‐transfected cells. A) EndoC‐*β*H1 cells were left non‐transfected (NT) or transfected with PIC for 24 h and RNA immunoprecipitation was performed using a CTCF antibody or an IgG antibody used as a negative control. The input represents the amount of CTCF present in the lysate before the immunoprecipitation was performed. GAPDH was analyzed as a negative control for the immunoprecipitation. The image is representative of three independent experiments. B) *ARGI* expression was determined in CTCF‐bound RNA (white bars) or IgG‐bound RNA (grey bars) by qPCR. Results are means ± SEM of three independent experiments and the amounts of *ARGI* are expressed as relative to input. **p < 0.01, and *p < 0.05 as indicated; ANOVA followed by Bonferroni's multiple comparisons test. C–F) Chromatin RNA immunoprecipitation was performed in non‐transfected (NT) and PIC‐transfected HEK293 cells. CTCF‐bound chromatin and RNA were immunoprecipitated using an antibody for CTCF (white) or IgG (grey), used as negative control. CTCF‐bound *ARGI* expression (C), *IFNβ* promoter (D), *ISG15* promoter (E), and *ISG15* enhancer amounts (F) were determined by qPCR. Results are means ± SEM of three independent experiments and are presented as relative to the input. ***p < 0.001, **p < 0.01, and *p < 0.05 as indicated; ANOVA followed by Bonferroni's multiple comparisons test.

The regulatory regions (promoters and enhancers) of *IFNβ* and *ISG15* contain binding sites for several key pro‐inflammatory transcription factors (IRF7, STAT1, and STAT2, among others). In addition, there are binding sites for CCCTC‐binding factor (CTCF), a conserved zinc finger protein that can act as a transcriptional activator, repressor, or insulator protein, blocking the communication between enhancers and promoters.^[^ ^37^, ^38^ ^]^ *In silico* prediction of RNA‐protein interactions using the CatRAPID tool revealed that *ARGI* potentially interacts with CTCF (interaction score: 0.41), but not with STAT1, STAT2, or IRF7 proteins.^[^ ^39^ ^]^ To experimentally assess whether *argi* interacts with CTCF, we performed RNA immunoprecipitation using a CTCF‐targeting antibody in basal and PIC‐transfected pancreatic *β* cells (**Figure** **5**A). Under basal conditions, *argi* and CTCF did not interact, but upon exposure to intracellular PIC, *argi* bound to CTCF in pancreatic *β* cells (Figure 5B).

To check whether *argi* and CTCF in turn interact with *IFNβ* and *ISG15* regulatory regions, we next performed a chromatin‐RNA immunoprecipitation to immunoprecipitate RNA and chromatin fragments simultaneously bound to CTCF. To this end, CTCF‐bound chromatin and RNA were captured using an anti‐CTFC antibody, and *ARGI*, *IFNβ* promoter, and *ISG15* promoter and enhancer were amplified by qPCR. As shown in Figure 5C–F, CTCF was bound to *ARGI* and simultaneously interacted with *IFNβ* promoter, *ISG15* promoter and enhancer, especially upon PIC transfection.

### 2.5 Allele‐Specific Binding of *ARGI* to CTCF Affects IDIN Gene Expression Levels

**Figure 6:**
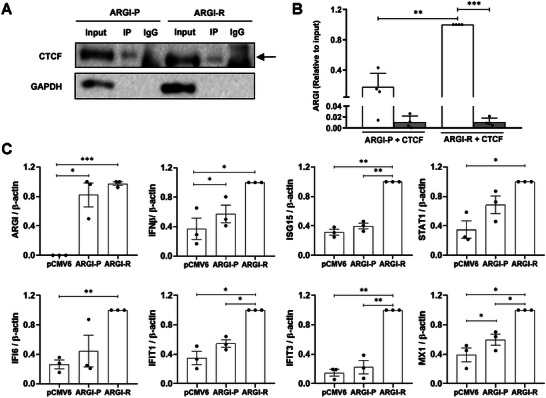
Allele‐specific binding of *ARGI* to CTCF affects IDIN gene expression. A) EndoC‐*β*H1 cells were transfected with a plasmid overexpressing *ARGI* harboring the T1D protective (ARGI‐P) or risk allele (ARGI‐R) and co‐transfected with a vector overexpressing CTCF. RNA immunoprecipitation was performed using an antibody for CTCF; IgG was used as negative control. The input represents the amount of CTCF present in the lysate before the immunoprecipitation was performed. GAPDH was analyzed as a negative control for the immunoprecipitation. The image is representative of four independent experiments. B) CTCF‐ (white bars) and IgG‐bound (grey bars) *ARGI* amounts were determined by qPCR. Results are means ± SEM of 4 independent experiments and the amounts of *ARGI* are presented as relative to the input. ***p < 0.001 and **p < 0.01 as indicated; ANOVA followed by Bonferroni's multiple comparisons test. C) EndoC‐*β*H1 cells were transfected with control plasmid (pCMV6) or plasmids overexpressing *ARGI* harboring the protective (ARGI‐P) or risk allele (ARGI‐R). Expression of *ARGI*, *IFNβ, ISG15, STAT1, IFI6, IFIT1, IFIT3*, and *MX1* was determined by qPCR and normalized to the reference gene *β*‐actin. Results are means±SEM of 3 independent experiments and are presented as relative to the input. ***p < 0.001, **p < 0.01, and *p < 0.05 as indicated; ANOVA followed by Bonferroni's multiple comparisons test.

LncRNA secondary structure is crucial for its function since it may affect its stability and binding to DNA, proteins, or other RNAs.^[^ ^40^, ^41^, ^42^, ^43^ ^]^ *argi* harbors a T1D‐associated SNP that was predicted to affect its secondary structure (Figure S2, Supporting Information). To test whether the T1D‐associated SNP affects *argi* function, we first determined the interaction of *argi* and CTCF in the presence of the T1D protective or risk allele. To this end, we performed RNA immunoprecipitation in cells overexpressing CTCF and *argi* harboring the T1D protective (argi‐P) or risk allele (argi‐R) (**Figure** **6**A). As shown in Figure 6B, *argi* containing either allele interacted with CTCF, but the interaction was stronger when *argi* harbored the T1D risk allele (rs9585056‐G) than when it had the protective allele (rs9585056‐A).

We next analyzed the expression of *IFNβ*, *ISG15*, and other IDIN genes in EndoC‐*β*H1 cells overexpressing argi‐P or argi‐R (Figure 6C). Both vectors induced similar *argi* expression, suggesting that the T1D‐associated SNP did not affect *argi* stability. Interestingly, overexpression of *argi* harboring the T1D risk allele induced a higher expression of *ISG15*, *IFIT1, IFIT3*, and *MX‐1* genes than overexpression of *argi* harboring the T1D protective allele (Figure 6C). Overexpression of both, ARGI‐P and ARGI‐R, increased *IFNβ*  expression when compared to pCMV6‐transfected EndoC‐*β*H1 cells, and ARGI‐R upregulation induced a higher expression of *STAT1* and *IFI6* genes when compared to pCVM6‐transfected cells, that was not observed in ARGI‐P‐overexpressing cells. In summary, these results confirm that upregulation of *ARGI* harboring the T1D risk allele (ARGI‐R), induces a higher transcription of some ISGs in comparison with ARGI‐P‐overexpressing *β* cells.

## 3 Discussion

T1D is a complex autoimmune disease in which genetic and environmental factors interact to trigger an autoimmune assault against pancreatic *β* cells.^[^ ^2^ ^]^ Several *loci* throughout the human genome have been associated with genetic risk for T1D, and several candidate genes have been proposed as being causal. Many of the T1D candidate genes characterized so far have been implicated in the regulation of antiviral and pro‐inflammatory responses at the pancreatic *β* cell level.^[^ ^44^ ^]^ Some participate in the regulation of the type I IFN signaling, such as *PTPN2* and *TYK2*, by regulating the type I IFN‐induced JAK/STAT signaling pathway,^[^ ^3^, ^4^, ^45^ ^]^ while others (e.g., *MDA5*) encode viral dsRNA cytoplasmic receptors.^[^ ^6^ ^]^ The interaction between genetic variants in T1D candidate genes and viral infections have been studied these past years, demonstrating that T1D risk alleles hyperactivate antiviral and pro‐inflammatory responses in pancreatic *β* cells, that eventually lead to *β* cell destruction and T1D development.^[^ ^45^, ^46^, ^47^, ^48^ ^]^

Over the past years, advances in the annotation of the human genome have revealed that many disease‐associated SNPs are located within lncRNAs, affecting their function by disrupting their secondary structure.^[^ ^24^ ^]^ For instance, the T1D‐associated lncRNA *Lnc13* regulates virus‐induced STAT1 signaling in pancreatic *β* cells in an allele‐specific manner.^[^ ^5^ ^]^ Another lncRNA that harbors an SNP associated with T1D is *NONHSAG044354*, a lncRNA that is located over the T1D candidate gene *BACH2*.^[^ ^23^ ^]^ Even though its function has not been characterized in pancreatic *β* cells yet, this T1D‐associated lncRNA might regulate the expression of BACH2, a transcription factor that has been shown to participate in cytokine‐induced pancreatic *β* cell apoptosis.^[^ ^49^ ^]^ Another lncRNA studied in the context of T1D is *MEG3*. This RNA molecule binds directly to EZH2, resulting in the upregulation of MafA and increasing the synthesis and secretion of insulin.^[^ ^50^ ^]^ LncRNA *MALAT1* induces *β* cell dysfunction via reduction of the histone acetylation of the PDX1 promoter that, in turn, suppresses insulin release.^[^ ^51^ ^]^

The function of most T1D‐associated lncRNAs has not been characterized. Several studies point to the implication of non‐coding RNA molecules in the regulation of pancreatic *β* cell function, both in health and disease. Indeed, RNAseq studies in pancreatic islets have revealed the presence of hundreds of lncRNAs that are specifically expressed in pancreatic islets. Many of them are regulated by diabetogenic stimuli, such as pro‐inflammatory cytokines, suggesting a potential role in the regulation of pro‐inflammatory pathways in pancreatic *β* cells.^[^ ^52^, ^53^, ^54^ ^]^

The implication of lncRNAs in the regulation of pro‐inflammatory pathways has also been documented in other contexts. For example, the lncRNA *LUCAT1* is a negative feedback regulator of IFN responses in humans by interacting with STAT1 in the nucleus,^[^ ^55^ ^]^ and lncRNA *Mirt2* inhibits activation of NF*κ*B and MAPK pathways, limiting the reduction of LPS‐induced proinflammatory cytokines, through attenuation of Lys63 (K63)‐linked ubiquitination of TRAF6 in macrophages.^[^ ^56^ ^]^

Herein, we functionally characterized the lncRNA *argi* (Antiviral Response Gene Inducer) in the regulation of virus‐induced pancreatic *β* cell inflammation. The SNP associated with T1D (rs9585056) was initially cataloged as an intergenic SNP that correlated with the expression of the antiviral gene network IDIN.^[^ ^29^ ^]^ IDIN is enriched with ISGs, such as *ISG15*, *MX1*, or *IF6*, among others. Our analysis showed that the T1D‐associated rs9585056 is actually located in an exon of *argi*, and *in silico* predictions revealed that the SNP disrupts its secondary structure.

RNA‐sequencing experiments of *argi*‐overexpressing pancreatic *β* cells revealed a role for *argi* in antiviral and pro‐inflammatory gene expression regulation, with upregulation of type I IFN signaling and antiviral pathways. IDIN genes were significantly overrepresented among the genes induced by *argi*‐overexpression, suggesting that *argi* may regulate IDIN in pancreatic *β* cells.

Our data revealed that *ARGI* does not participate in virus‐induced pancreatic *β* cell death. These results are in line with observations for other T1D candidate genes. For example, the T1D candidate gene *MDA5* is implicated in virus‐induced NF*κ*B activation and pro‐inflammatory gene expression in pancreatic *β* cells, but it does not participate in viral dsRNA‐induced apoptosis.^[^ ^6^ ^]^ These results suggest that *ARGI* might participate in the initial stages of T1D, promoting the inflammatory environment that precedes pancreatic *β* cell destruction.

Importantly, our data showed that allele‐specific upregulation of *argi* in pancreatic *β* cells led to increased expression of IDIN genes in an allele‐specific manner. *argi* harboring the T1D risk allele induced a higher expression of IDIN genes than the lncRNA harboring the T1D protective allele. Since the secondary/tertiary structure of lncRNAs seems to be crucial for their function, genetic variants, and mutations potentially contribute to disease pathogenesis by altering disease‐associated pathways.^[^ ^5^, ^24^, ^57^ ^]^ We found that *argi* regulates the expression of some ISGs in an allele‐specific manner through its interaction with the transcription factor CTCF. Viral dsRNA triggered translocation of *argi* to the nucleus of pancreatic *β* cells. Once in the nucleus, *argi* interacts with CTCF to bind to regulatory regions of some ISGs (e.g., *IFNβ* and *ISG15*) to transcriptionally activate them. Also under basal conditions, the interaction between CTCF and *argi* is stronger for the T1D risk allele than for the protective allele, suggesting that stronger binding between *argi* and CTCF will exacerbate ISG expression in pancreatic *β* cells. Other studies have shown that interaction between disease‐associated lncRNAs and transcription factors, both at the mRNA and protein level, may affect gene expression in an allele‐specific manner. Indeed, *Lnc13* in pancreatic *β* cells interacts in an allele‐specific manner with PCBP2 to stabilize *STAT1* mRNA, leading to increased STAT1 activation and pro‐inflammatory chemokine expression when the T1D risk allele is present in *Lnc13*.^[^ ^5^ ^]^ Along the same lines, a lncRNA harboring an SNP associated with cancer (*CCAT2*) regulates cancer cell metabolism through its interaction with the Cleavage factor I (CFIm) in an allele‐specific manner.^[^ ^58^ ^]^

The transcription factor CTCF is a ubiquitously expressed multifunctional transcriptional regulator. It can inhibit or activate gene transcription depending on the cell type, location of the binding site, stimulus, and presence of interacting partners (transcriptional activators, repressors, cohesins, and RNA pol II).^[^ ^59^ ^]^ Using RNA antisense purification and RNA immunoprecipitation, we observed that in the presence of viral dsRNA, *argi* and CTCF interact with the regulatory regions of *IFNβ* and *ISG15*, suggesting that viral infections in *β* cells promote ISG expression through the binding of *argi* and CTCF to regulatory regions of these antiviral genes. Recent studies have shown that CTCF can bind non‐coding RNAs to modulate gene expression, including inflammation‐related genes.^[^ ^60^, ^61^ ^]^ Using RedCHIP technique, a recent study identified several *cis*‐ and *trans*‐acting non‐coding RNAs enriched at CTCF‐binding sites in human cells, which may be involved in CTCF‐dependent chromatin looping and gene activation/repression.^[^ ^60^ ^]^ Some studies have characterized the impact of CTCF and non‐coding RNA interaction in the regulation of inflammatory genes. For example, a non‐coding RNA derived from normally silenced pericentromeric repetitive sequences was shown to impair CTCF binding to chromatin, resulting in the alteration of chromatin accessibility and the activation of SASP‐like inflammatory genes.^[^ ^61^ ^]^

One of the main limitations of the present study is the use of an immortalized human pancreatic *β* cell line (EndoC‐*β*H1 cells) to perform most of the functional in vitro experiments. Primary human islets or purified primary human pancreatic *β* cells are the gold standard for in vitro diabetes modeling; however, they are difficult to obtain and isolate, limiting their use in in vitro studies, especially when high amounts of cells are needed.^[^ ^62^, ^63^ ^]^ The use of iPSC‐derived human islet‐like aggregates constitutes a useful and disease‐relevant alternative to human islets to perform in vitro studies in diabetes.^[^ ^64^, ^65^, ^66^ ^]^ However, some genetic manipulations are particularly inefficient in these cells such as plasmid‐mediated protein overexpression and gene silencing of very low expressed gene targets, as is the case for lncRNAs.^[^ ^63^, ^67^ ^]^ In the context of this manuscript, the technical issues associated with the genetic manipulation of iPSC‐derived human islet‐like aggregates impeded the validation of some results obtained in EndoC‐*β*H1cells, pointing to the future need to fine tune these in vitro approaches and to develop novel techniques to efficiently modify the expression of low abundance non‐coding transcripts.

In the meantime, the use of the EndoC‐*β*H1 cell line has been proven as a valid human pancreatic *β* cell model, not only for the characterization of T1D‐associated genes, but also for the study of *β* cell physiology or for screenings to identify novel drug target candidates.^[^ ^68^, ^69^, ^70^ ^]^ Moreover, the EndoC‐*β*H1 cells open chromatin, transcriptomics, and miRNA landscapes have been reported to be similar to adult human *β* cells or islets, further supporting that they are good models of human pancreatic *β* cells.^[^ ^71^ ^]^

**Figure 7:**
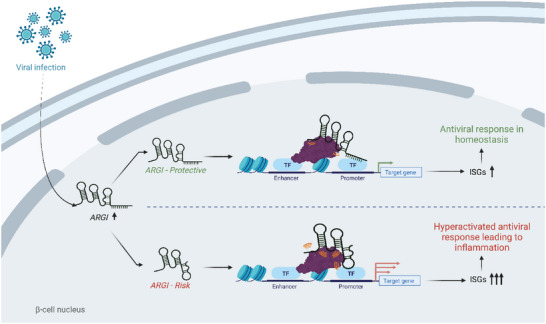
A model of the role of *ARGI* in the allele‐specific modulation of virus‐induced ISG transcription in pancreatic *β* cells. Viral infections trigger *ARGI* upregulation in the nuclei of pancreatic *β* cells. *ARGI* interacts with the transcription factor CTCF to bind to the regulatory regions of interferon‐stimulated genes (ISGs), promoting their transcription. When *ARGI* harbors the T1D protective allele, a homeostatic antiviral response is activated; however when the T1D risk allele is present, this process is exacerbated, leading to a hyperactivation of the antiviral response in pancreatic *β* cells. In the context of T1D pathogenesis, the generation of an excessive pro‐inflammatory environment in pancreatic islets, leads to increased insulitis, and eventually, to pancreatic *β* cell destruction and T1D development.

In summary, our data demonstrate that *argi* is implicated in the regulation of virus‐induced inflammation in pancreatic *β* cells through a molecular mechanism that implies allele‐specific binding to the transcription factor CTCF and regulation of ISG expression, a gene expression signature related to the T1D pathogenesis (**Figure** **7**). Our results are in concordance with the expression of type I IFNs in pancreatic islets of T1D donors.^[^ ^72^, ^73^, ^74^ ^]^ Moreover, more recent studies, such as the Diabetes Virus Detection (DiViD) study, have observed upregulation of several ISGs, including *STAT1*, *IFI6*, or *MX1* in islets from individuals with T1D.^[^ ^48^, ^75^ ^]^

## 4 Conclusion

Overall, our data demonstrate the implication of a T1D‐associated lncRNA in the regulation of antiviral and pro‐inflammatory responses in pancreatic *β* cells. The implication of viral infections and the resulting activation of type I IFN signaling in *β* cells seem to be crucial in the initial stages of T1D. However, the specific pathogenic mechanisms that trigger the infection‐induced autoimmune assault against *β* cells are not fully elucidated. Accumulating evidence suggests that the factor linking viral infections with the autoimmune triggering rely on the presence of T1D‐associated genetic variants in genes expressed in pancreatic *β* cells.^[^ ^3^, ^4^, ^5^, ^6^ ^]^ Hence, the functional characterization of T1D susceptibility genes in virus‐induced pancreatic *β* cell dysfunction will help to clarify many of the “unknowns” that still linger regarding the pathogenesis of the disease. In this sense, our findings provide novel information on the molecular mechanisms by which disease‐associated SNPs in lncRNAs influence the activation of diabetogenic gene expression pathways in pancreatic *β* cells, and on how interactions between T1D‐associated genes and infections may trigger inflammation in the initial stages of the disease.

## 5 Experimental Section

### Reagents, Tools, and Datasets

All the reagents, tools, and datasets used in the present study are listed in Table S1 (Supporting Information).

### Human Cell Lines

The EndoC‐*β*H1 human pancreatic *β* cell line was purchased from Human Cell Design (https://www.humancelldesign.com). Cells were cultured and split as previously described.^[^ ^76^ ^]^ Briefly, EndoC‐*β*H1 cells were seeded at an approximate density of 70,000‐75,000 cells cm^−2^ on culture plates pre‐coated with Matrigel‐fibronectin (100 mg L^−1^ and 2 mg mL^−1^, respectively, Sigma–Aldrich) at 37 °C, 5% CO_2_ in complete OPTI*β*1 medium (Human Cell Design). Cells were passed every 7 days. For transfection, DMEM medium was used containing 2% FBS, 5.6 mmol L^−1^ glucose, 50 µmol L^−1^ 2‐mercaptoethanol (Biorad, CA, USA), 10 mmol L^−1^ nicotinamide (Calbiochem, Darmstadt, Germany), 5.5 µg mL^−1^ transferrin, 6.7 ng mL^−1^ selenite (Sigma–Aldrich).

HEK293 cells (CRL‐1573) were purchased from the American Type Culture Collection (ATCC; https://www.atcc.org). Cells were cultured in DMEM supplemented with 10% FBS and 100 units mL^−1^ penicillin and 100 µg mL^−1^ streptomycin (Lonza). The same medium without antibiotics was used for transfection.

### Human iPSC‐Derived Islet‐Like Aggregates

Two human iPSC lines (HEL115.6 and 1023A) derived from healthy donors were cultured and differentiated into *β* cells as described previously. HEL115.6 iPSCs were generated at the University of Helsinki; 1023A iPSCs were kindly provided by Dr DM Egli (Columbia University).^[^ ^66^ ^]^ The iPSCs had normal karyotype, stem cell colony morphology, and expressed pluripotency markers.^[^ ^66^, ^77^ ^]^ iPSCs were maintained in E8 medium on Matrigel‐coated plates (Corning) and seeded at 2.5–2.8 × 10^6^ cells per 3.5 cm well in E8 medium containing 5 µmol L^−1^ ROCK inhibitor (STEMCELL Technologies) 24 h prior to the 7‐stage differentiation. When reaching pancreatic progenitor stage, cells were plated into 24 well microwell plates at a density of 750 cells per microwell (AggreWell, STEMCELL Technologies) with 10 µmol L^−1^ ROCK inhibitor and 1 µmol L^−1^ heparin (STEMCELL Technologies) to allow 3D formation of aggregates. Prior to infection, stage 7 aggregates were dispersed. Aggregates were incubated in 0.5 mmol L^−1^ EDTA at room temperature for 4 min, exposed to Accumax (Sigma–Aldrich) for 8 min and then dispersed by gentle pipetting.^[^ ^78^ ^]^ Knockout serum (Gibco) with 10 µm ROCK inhibitor was added to quench the dissociation process, and cells were seeded at 5 × 10^4^ cells per 6.4 mm well in stage 7 medium.^[^ ^66^ ^]^

### PIC Transfection, Viral Infection, and Cell Treatments

The synthetic viral dsRNA mimic PIC (InvivoGen) was used at a concentration of 0.25 µg mL^−1^ and transfected using Lipofectamine‐2000 (Invitrogen) as previously described.^[^ ^3^ ^]^

For viral infection, human iPSC‐derived islet‐like aggregates were dispersed using Accumax (Sigma) as previously described.^[^ ^65^, ^78^ ^]^ Cells were plated at a cell density of 300,000 cell per well in Matrigel‐coated 24‐well plates in stage 7 medium supplemented with 10 µmol L^−1^ ROCK inhibitor.^[^ ^66^ ^]^ After overnight recovery, cells were infected with CVB1/Conn‐5 (MOI = 0.05) and CVB4/JVB (MOI = 0.5) in Ham's F‐10 Nutrient Mixture (Gibco), supplemented with 2 mmol L^−1^ GlutaMAX, 50 µmol L^−1^ IBMX and 1% FBS. Two hours after infection medium was replaced by Ham's F‐10 Nutrient Mixture, 0.75% BSA, 2 mmol L^−1^ GlutaMAX, 50 µmol L^−1^ IBMX, 50 U mL^−1^ penicillin, 50 µg mL^−1^ streptomycin, and cells were cultured for an additional 22 h as previously described.^[^ ^31^ ^]^

The NF*κ*B inhibitor Bay 11–7082 (Sigma‐Aldrich) was used at a concentration of 10 µmol L^−1^.

### RNA Extraction and Quantitative PCR

RNA extraction was performed using the NucleoSpin RNA Kit (Macherey Nagel), PureLink RNA Mini kit (Invitrogen), or miRNeasy mini kit (Qiagen), and expression values were determined by qPCR using Taqman Gene Expression Assays (Thermo Scientific) specific for *ARGI, MX1, ISG15, IFI6, IFIT1, IFIT3, STAT1, MEG3, RPLP0*, and *IFNβ* or by Sybr Green (Biorad) using specific primers for *ARGI, ISG15* promoter, *ISG15* enhancer, and *IFNβ* promoter. The Taqman Gene Expression Assays and primers are listed in Table S1 (Supporting Information).

For the determination of gene expression in 12 different human tissues, the Human total RNA master panel II (Clontech) was employed at a final concentration of 10 ng µL^−1^ of RNA.

qPCR measurements were performed in duplicate in at least three independent samples and expression levels were analyzed using the 2^−ΔΔCt^ method.

### Cellular Fractionation

For quantification of *ARGI* RNA levels in whole cell and nuclear extracts, nuclei were isolated using C1 lysis buffer (1.28 mol L^−1^ sucrose, 40 mmol L^−1^ Tris‐HCl pH 7.5, 20 mmol L^−1^ MgCl2, 4% Triton X‐100). *ARGI*, *MEG3*, *or MALAT1* (as nuclear controls), and *RPLP0* (cytoplasmic control) levels were quantified by qPCR and compared to the total amount of those RNAs in the whole cell lysate.

### RNA‐Sequencing

RNA‐sequencing of control and ARGI‐overexpressing pancreatic *β* cells was performed in a NovaSeq 6000 sequencer. Sequencing libraries were prepared using “TruSeq Stranded Total RNA Human” kit (Illumina Inc., Cat.# RS‐122‐2201), following “TruSeq Stranded Total RNA Sample Prep‐guide (Part # 15 031 048 Rev. E)”.

Reads were trimmed using Trimmomatic 0.39, with default settings. Then, reads were aligned by means of HISAT2 using as reference Human Genome assembly hg38, as it is available from the developer site. Stringtie was used to calculate transcripts and their abundances; and htseq‐count to get the counts of each transcript.

Transcripts with more than 10 counts in at least three samples were kept. Then, counts were adjusted by means of RUVSeq package of R language, and *C1orf43*, *EMC7*, *PSMB2*, *PSMB4*, *RAB7A*, *REEP5*, and *VPS29* genes were used as negative controls in the estimation of the factors of unwanted variation. edgeR package in R language was used to calculate differential expression between control and *ARGI* overexpression, using the upper quartile method for normalization and considering that samples were paired. Gene‐set enrichment analyses were carried out by means of enrichR, and additional statistics analyses and graphs were made using R language base functions, ggplot2 package, and cyclize package.

### RNAseq data from primary human pancreatic *α* and *β* cells

For the determination of *ARGI* expression in human *α* and *β* pancreatic cells, raw FASTQ files corresponding to 7 *α* cell RNAseq samples and 8 *β* cell RNAseq samples were downloaded from the GEO database (GSE76268). Single end reads were pseudo‐aligned to hg38 using Kallisto 0.46.1 with default parameters. Transcript level counts were summarized per gene using tximport function in R 4.2.2.

### Western blot analysis

EndoC‐*β*H1 cells were washed with cold PBS and lysed in Laemmli buffer (62 mmol L^−1^ Tris‐HCl, 100 mmol L^−1^ dithiothreitol (DTT), 10% vol/vol glycerol, 2% wt/vol SDS, 0.2 mg mL^−1^ bromophenol blue, 5% vol/vol 2‐mercaptoethanol). Proteins in the lysate were separated by SDS‐PAGE. Following electrophoresis, proteins were transferred onto nitrocellulose membranes using a Transblot‐Turbo Transfer System (Bio‐Rad) and blocked in 5% wt/vol non‐fatty milk diluted in TBST (20 mmol L^−1^ Tris, 150 mmol L^−1^ NaCl and 0.1% vol/vol Tween 20) at room temperature for 1 h. The membranes were incubated overnight at 4 °C with a primary antibody specific for CTCF (Cat #PA5‐17143, Invitrogen) diluted 1:1,000 in 5% wt/vol BSA or anti‐GAPDH (SC‐365062, Santa Cruz Biotechnologies) diluted 1:5,000 in 5% wt/vol BSA. Immunoreactive bands were revealed using the Clarity Max Western ECL Substrate (Bio‐Rad) after incubation with a horseradish peroxidase‐conjugated anti‐rabbit (1:1,000 dilution in 5% wt/vol non‐fatty milk) or anti‐mouse (1:5,000 dilution in 5% wt/vol non‐fatty milk) secondary antibody for 1 h at room temperature. The immunoreactive bands were detected using a Bio‐Rad Molecular Imager ChemiDoc XRS and quantified using ImageLab software (Bio‐Rad).

### ARGI Overexpression and Silencing Experiments

The overexpression vector for *ARGI* harboring the T1D risk allele (ARGI‐R) was purchased from ProteoGenix (Schiltigheim, France). The overexpression vector for *ARGI* harboring the T1D protective allele (ARGI‐P) was produced by site‐directed mutagenesis using the Site‐Directed Mutagenesis QuickChange II (Agilent). *ARGI*‐overexpressing plasmids were transfected using Lipofectamine‐2000 (Invitrogen) following the manufacturer's instructions.

*ARGI* was silenced by transfection of two different small interfering (si)RNAs (Table S1, Supporting Information) using Lipofectamine RNAi Max (Invitrogen) following the manufacturer's instructions.

### Caspase 3/7 Activation

The analysis of caspase 3/7 activation was performed using the Caspase‐Glo 3/7 Assay from Promega, following the manufacturer's instructions.

### CRISPRi and CRISPR‐Cas9 Experiments

For CRISPR‐Cas9 experiments, sgRNAs (Table S1, Supporting Information) were designed using the Zhang lab tool (currently Synthego) and cloned into the pX330 vector (Addgene) using BbsI restriction site (NEB). Plasmids harboring the sgRNAs were transfected into cells using Lipofectamine‐2000. PIC was transfected as described above 3 days post sgRNA transfection.

For CRISPRi experiments a sgRNA (Table S1, Supporting Information) was cloned into a CRISPRi vector harboring the dCas9 protein (Origene) using BamHI and BsmBI restriction sites. The plasmid harboring the sgRNA was transfected into cells using Lipofectamine‐2000 and PIC was transfected as described above 3 days post sgRNA transfection.

### RNA Immunoprecipitation Assay

For RNA immunoprecipitation experiments, EndoC‐*β*H1 cells were kept untransfected or transfected with 0.25 µg mL^−1^ of PIC using Lipofectamine‐2000 for 24 h. Cells were lysed in RNA immunoprecipitation buffer (150 mmol L^−1^ KCl, 25 mmol L^−1^ Tris, 0.5 mmol L^−1^ DTT, 0.5% NP‐40, protease inhibitors), kept on ice for 15 min and homogenized using a syringe. Lysates were pre‐cleared with Dynabeads G (ThermoFisher) for 1 h in a wheel shaker at 4°C. Before immunoprecipitation was performed, 10% of each lysate was saved as input. The remaining pre‐cleared lysates were incubated with an anti‐IgG antibody (negative control; Santa Cruz Biotechnologies) or anti‐CTCF antibody (ThermoFisher) for 1 h at room temperature in a wheel shaker. After, Dynabeads were added and the mix further incubated for 30 min in the wheel shaker. Supernatants were removed and beads washed three times with RNA immunoprecipitation buffer, three times with low salt buffer (50 mmol L^−1^ NaCl, 10 mmol L^−1^ Tris‐HCl, 0.1% NP‐40), three times with high salt buffer (500 mmol L^−1^ NaCl, 10 mmol L^−1^ Tris‐HCl, 0.1% NP‐40), and then resuspended in RNA extraction buffer.

Western blot analysis was performed using specific antibodies for the protein of interest (CTCF) and GAPDH was analyzed as a negative control for the immunoprecipitation. All quantifications were normalized by the input using the following formula:

(1) $$\begin{array}{c} ARGI\;\mathrm{expression}\;\left(\mathrm{relative}\;\mathrm{to}\;\mathrm{input}\right)=100*2\wedge \left[\mathrm{Input}-\mathrm{IPCTCF}\;\mathrm{or}\;\mathrm{IPIgG}\right] \end{array}$$

The same procedure was used for immunoprecipitation of CTCF‐bound *ARGI* in ARGI‐R‐ or ARGI‐P‐ and CTCF‐overexpressing cells.

### RNA Antisense Purification

RNA antisense purification was performed following a protocol from the Guttman lab.^[^ ^79^ ^]^ In brief, probes were generated by in vitro transcribing biotinylated antisense *ARGI* followed by controlled RNA fragmentation using a RNA Fragmentation Reagent (Invitrogen). Probes against a non‐related lncRNA of similar size were used as control. Extracts from EndoC‐*β*H1 cells were crosslinked adding 2 mm disuccinimidyl glutarate for 45 min at room temperature and subsequently 3% formaldehyde for 10 min at 37 °C. Crosslinked RNA‐chromatin was fragmented by sonication, hybridized with control or *ARGI*‐specific RNA probes, captured using Streptavidin Mag Sepharose beads (Cytiva), washed extensively, and eluted. Retrieved RNA was purified using PureLink Micro RNA columns (Thermo Scientific) and fragmented DNA by NucleoSpin Gel and PCR Clean‐up (Macherey‐Nagel). Enrichment of *ARGI* in the regulatory regions of IFN*β* and ISG15 was quantified by qPCR.

### Chromatin RNA Immunoprecipitation

HEK293 cells were transfected with PIC, fixed with disuccinimidyl glutarate and formaldehyde; chromatin was sheared by sonication and immunoprecipitated using protein G magnetic Dynabeads and CTCF antibody. Immunoprecipitated material was eluted, reverse crosslinked, and treated with Proteinase K. RNA was extracted using PureLink Micro RNA columns and DNA was extracted using the NucleoSpin Gel and PCR Clean‐up. Immunoprecipitated RNA and DNA analyzed by qPCR using primers specific for *ARGI* and regulatory regions of IFN*β* and ISG15.

HEK293 cells were used in this experiment because it requires 8–10 million cells per condition; the low proliferation rate of EndoC‐*β*H1 cells does not allow to obtain this large number of cells.

### Quantification and Statistical Analysis

Experimental data were normalized by the highest value in each experimental replicate (for a minimum of 3 samples) and displayed as means±SEM. Statistical analyses were performed with GraphPad Prism version 8. Two‐tailed Student's *t*‐test or ANOVA followed by Bonferroni's multiple comparisons tests were used to detect differences between two groups or more than two groups, respectively. *p*‐Values below 0.05 were considered statistically significant.

## Conflict of Interest

The authors declare no conflict of interest.

## Author Contributions

I.S. contributed to the original idea, designed the experiments, contributed to research data, and wrote the manuscript. I.G.M. contributed to research data and wrote the manuscript. K.G.E. and N.F.J. contributed to data analysis. L.M.M., J.M, A.O.G., M.N.A., T.S., C.M.C., C.V, A.O.B, M.C., and M.I.E. contributed to research data. M.C. edited the manuscript. All authors discussed the results, contributed to data interpretation, and approved the final manuscript

## Supporting information

Supporting Information

## Data Availability

The data that support the findings of this study are openly available in Gene Expression Omnibus at https://www.ncbi.nlm.nih.gov/geo/, reference number 217827.
