# Supplementary material for: LncRNA ARGI Contributes to Virus‐Induced Pancreatic β Cell Inflammation Through Transcriptional Activation of IFN‐Stimulated Genes
Source: Adv Sci (Weinh). 2023 Jun 29;10(25):2300063. doi: 10.1002/advs.202300063 (PMC10477904; doi:10.1002/advs.202300063)
Supplement: Supplementary file 1 — Supporting Information [file ADVS-10-2300063-s001.pdf]

ADVANCED SCIENCE
Open Access

Supporting Information

for *Adv. Sci.*, DOI 10.1002/advs.202300063

LncRNA *ARGI* Contributes to Virus-Induced Pancreatic β Cell Inflammation Through Transcriptional Activation of IFN-Stimulated Genes

*Itziar González-Moro, Koldo Garcia-Etxebarria, Luis Manuel Mendoza, Nora Fernández-Jiménez, Jon Mentxaka, Ane Olazagoitia-Garmendia, María Nicol Arroyo, Toshiaki Sawatani, Cristina Moreno-Castro, Chiara Vinci, Anne Op de Beek, Miriam Cnop, Mariana Igoillo-Esteve and Izortze Santin**

Supporting Information

LncRNA *ARGI* Contributes to Virus-Induced Pancreatic β Cell Inflammation Through Transcriptional Activation of IFN-Stimulated Genes.

Itziar González-Moro, *Koldo Garcia-Etxebarria*, *Luis Manuel Mendoza*, *Nora Fernández-Jiménez*, *Jon Mentxaka*, *Ane Olazagoitia-Garmendia*, *María Nicol Arroyo, Toshiaki Sawatani, Cristina Moreno-Castro, Chiara Vinci*, *Anne Op de Beek*, *Miriam Cnop*, *Mariana Igoillo-Esteve and Izortze Santin**.

I. Gonzalez-Moro, L.M. Mendoza, J. Mentxaka, A. Olazagoitia-Garmendia, I. Santin
Department of Biochemistry and Molecular Biology, University of the Basque Country, 48940 Leioa, Spain

I. Gonzalez-Moro, N. Fernandez-Jiménez, J. Mentxaka, A. Olazagoitia-Garmendia, I. Santin
Biocruces Bizkaia Health Research Institute, 48903 Barakaldo, Spain

K. Garcia-Etxebarria
Biodonostia Health Research Institute, Gastrointestinal Genetics Group, 20014 San Sebastián, Spain

K.Garcia-Etxebarria
Centro de Investigación Biomédica en Red de Enfermedades Hepáticas y Digestivas (CIBERehd), 08036 Barcelona, Spain

N. Fernández-Jiménez
Department of Genetics, Physical Anthropology and Animal Physiology, University of the Basque Country, 48940 Leioa, Spain

M.N. Arroyo, T. Sawatani, A., C. Moreno-Castro, C. Vinci, A. Op de Beek, M. Cnop, M. Igoillo-Esteve
ULB Center for Diabetes Research, Université Libre de Bruxelles, 1070 Brussels, Belgium

M. Cnop
Division of Endocrinology, Erasmus Hospital, Université Libre de Bruxelles, 1070 Brussels, Belgium

I. Santin
Centro de Investigación Biomédica en Red de Diabetes y Enfermedades Metabólicas Asociadas (CIBERDEM), Instituto de Salud Carlos III, 28029 Madrid, Spain

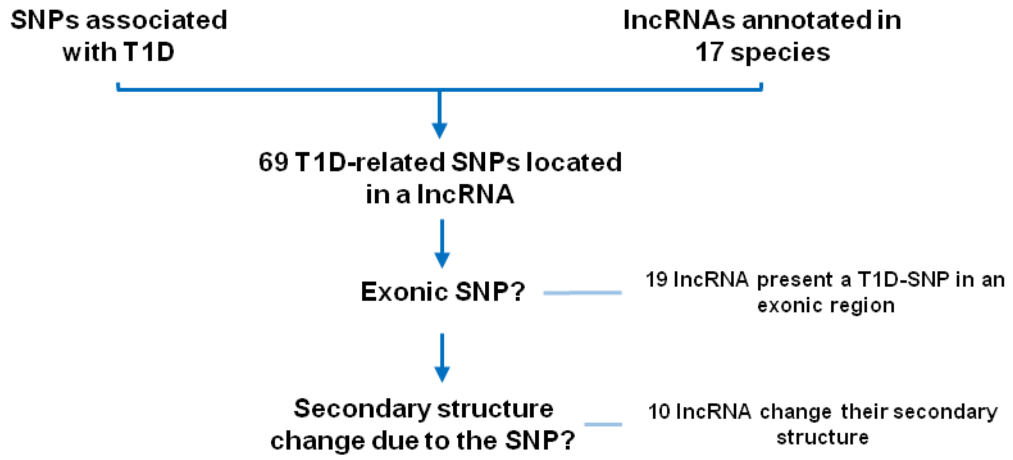

Figure S1. Schematic outline of the workflow used for T1D-associated lncRNA identification and selection. Genomic positions of T1D-associated SNPs annotated in the NHGRI-EBI Catalog of Human Genome Association Studies (EMBL-EBI) were intersected with the genomic localization of all lncRNAs annotated in NONCODE version 6. Nineteen T1D-associated lncRNAs harboring an exonic SNP were analyzed in ViennaRNA Web Services to determine potential changes in their secondary structure. Ten lncRNAs were predicted to undergo secondary structure changes due to the T1D-associated SNP.

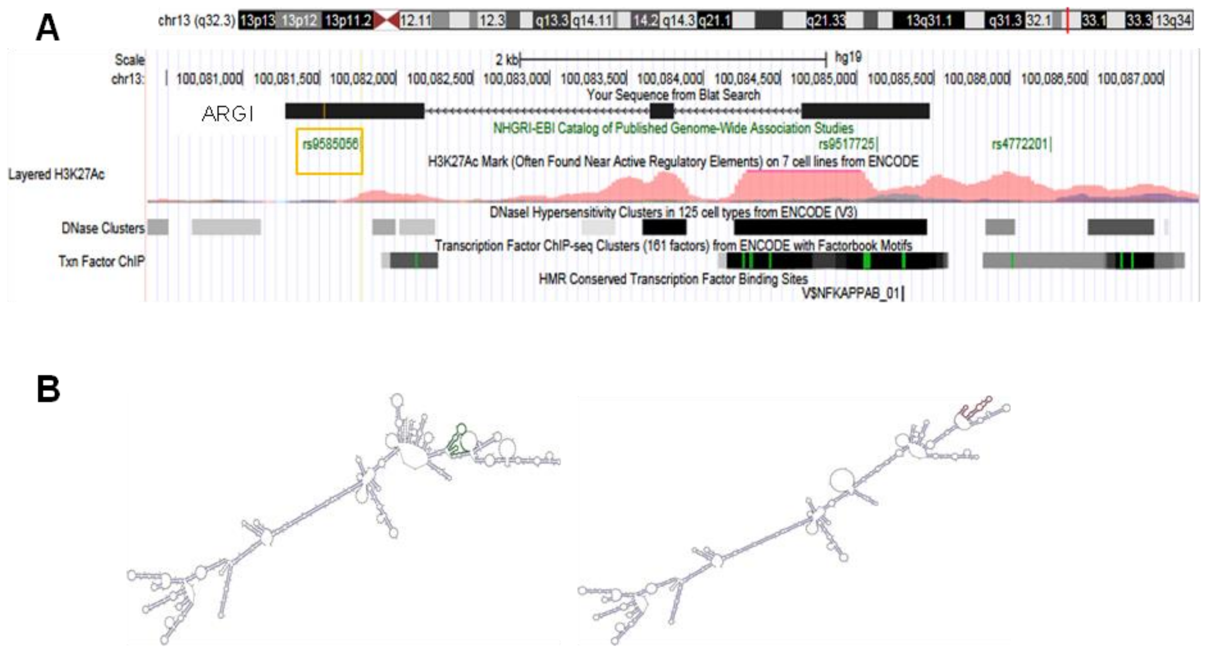

Figure S2. Genomic localization and secondary structure of *ARGI*. (A) *ARGI* is an intergenic lncRNA located in human chromosome 13 (99,429,023-99,433,220; GRCh38/hg38). It has three exons and harbors one T1D-associated SNP (rs9585056; chr13: 99,429,262-99) in its third exon (orange box). Epigenetic marks (H3K27Ac and DNase clusters) and a conserved NFkB binding site have been identified close to the transcription start site of *ARGI*. (B) Secondary structure of *ARGI* predicted using the ViennaRNA Web Services. The image shows the secondary structure of *ARGI* harboring the T1D protective (rs9585056-A; green) or risk allele (rs9585056-G; red). There is a significant difference in the *ARGI* secondary structure prediction for the risk allele compared to the protective one (p=0.0536; the software considers significant when p<0.2).

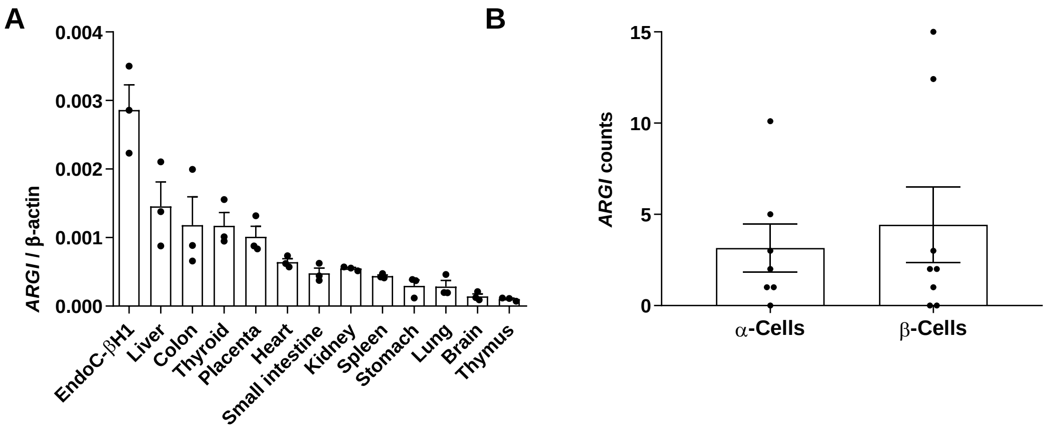

Figure S3. ***ARGI* is ubiquitously expressed in human tissues.** (A) *ARGI* expression was analyzed in the human β cell line EndoC-βH1 and in a set of human tissues (liver, colon, thyroid, placenta, heart, small intestine, kidney, spleen, stomach, lung, brain and thymus). *ARGI* expression was determined by qPCR and normalized to the reference gene β-actin. Results are means±SEM of 3 experimental replicates. (B) Expression of *ARGI* in human primary pancreatic α and β cells obtained from publicly available RNAseq data (GEO Omnibus; GSE76268). The relative expression level is represented as “Counts” and the expression data correspond to 7 α and 8 β cell samples.

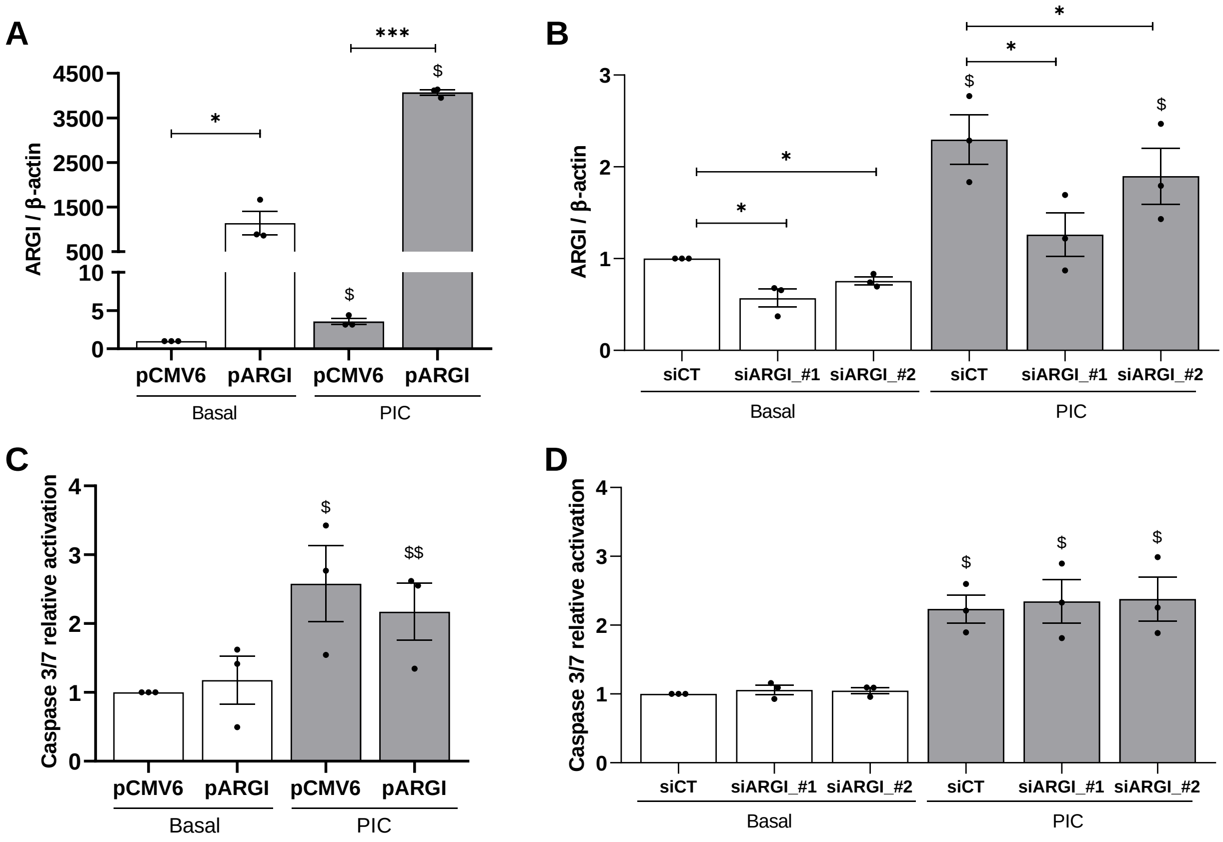

Figure S4. PIC-induced caspase 3/7 activation is not regulated by *ARGI* in EndoC-βH1 cells. *ARGI* was overexpressed (A) or silenced (B) in EndoC-βH1 cells using an overexpression vector or two specific siRNAs, respectively. *ARGI* expression was determined by qPCR and normalized by the reference gene β-actin. (C-D) PIC-induced Caspase 3/7 activation was measured in *ARGI*-overexpressing (C) or *ARGI*-silenced (D) EndoC-βH1 cells. Results are means±SEM of 3 independent experiments; \$ $p<0.05$ vs basal condition transfected with the same plasmid or siRNA; *** $p < 0.001$ and * $p < 0.05$ as indicated; ANOVA followed by Bonferroni's multiple comparisons test.

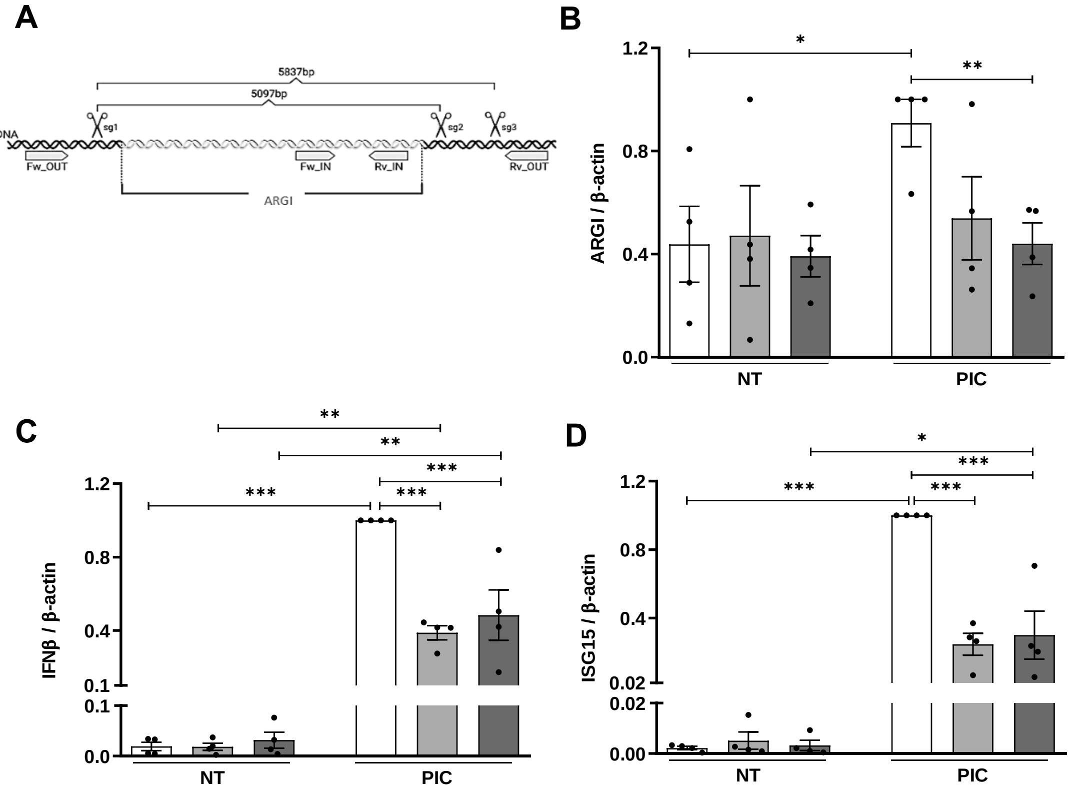

Figure S5. ***ARGI*** **disruption using CRISPR-Cas9 reduces PIC-induced** ***IFNβ*** **and** ***ISG15*** **expression.** (A) *ARGI* disruption was performed by generating a deletion of 5097 bp using single guide RNAs (sgRNAs) 1 and 2, or a deletion of 5837 bp using sgRNAs 1 and 3. The deletion was confirmed by PCR using a primer pair located inside the deleted region (for detection of unedited cells; wild type forward (Fw_IN) and wild type reverse (Rv_IN)) and a primer pair located outside the deleted region (for detection of edited cells; Fw_OUT and Rv_OUT). (B-D) EndoC-βH1 cells were transfected with an empty px330 vector (white bars) or with vectors harboring any of the two combinations of sgRNAs targeting *ARGI* (light and dark grey bars). After 36h, cells were left non-transfected (NT) or transfected with PIC (0.25 µg mL^{-1}) for 24h. Expression of *ARGI* (B), *IFNβ* (C) and *ISG15* (D) was determined by qPCR and normalized to the reference gene β-actin. Results are means±SEM of 4 independent experiments; ***p<0.001, **p <0.01 and *p <0.05 as indicated; ANOVA followed by Bonferroni's multiple comparisons test.

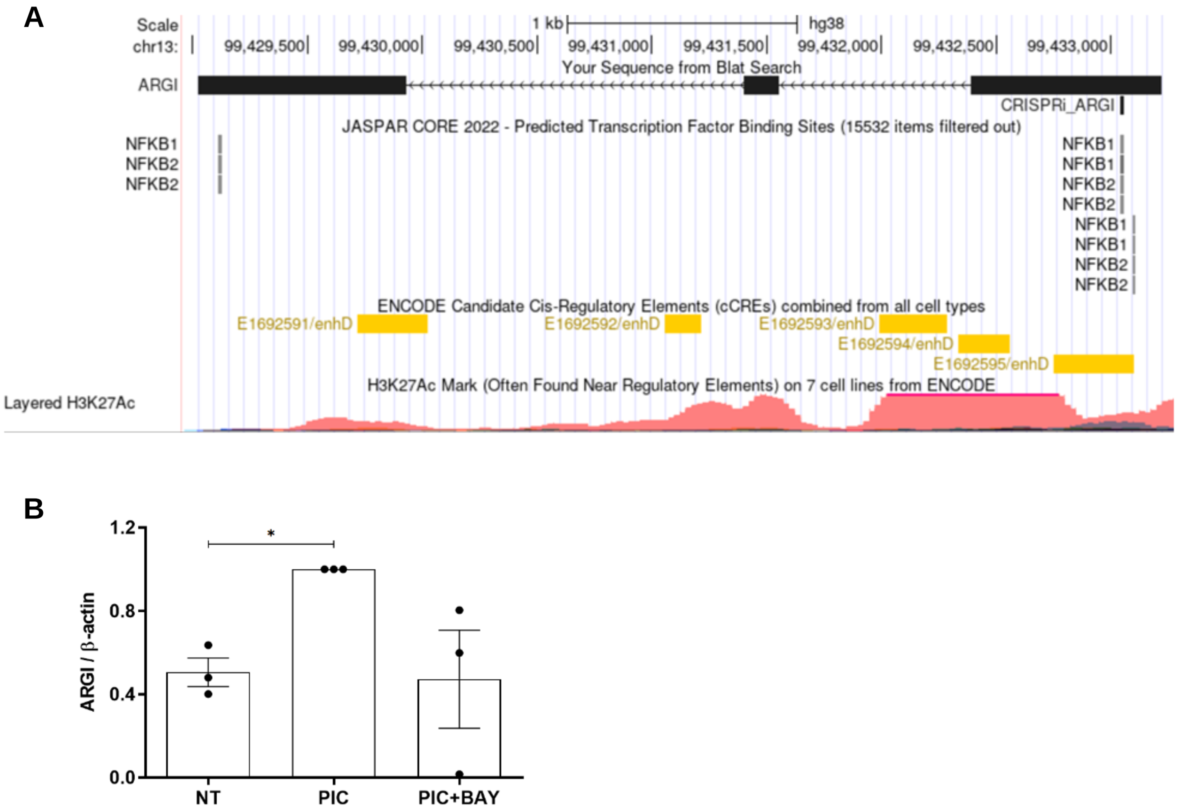

Figure S6. Inhibition of NFkB signaling counteracts PIC-induced *ARGI* upregulation in pancreatic β cells. (A) Genomic location of the CRISPRi guide designed for the inhibition of *ARGI*. The guide is complementary to a conserved NFκB binding site located close to the *ARGI* transcription start site. (B) Human EndoC-βH1 cells were left untreated (NT), treated with intracellular PIC (0.25 µg mL^{-1}) for 24h (PIC) or treated with PIC and Bay 11-7082 (PIC+BAY). *ARGI* expression was determined by qPCR and normalized to the reference gene β-actin. Results are means±SEM of 3 independent experiments; *p <0.05; Student's t test.

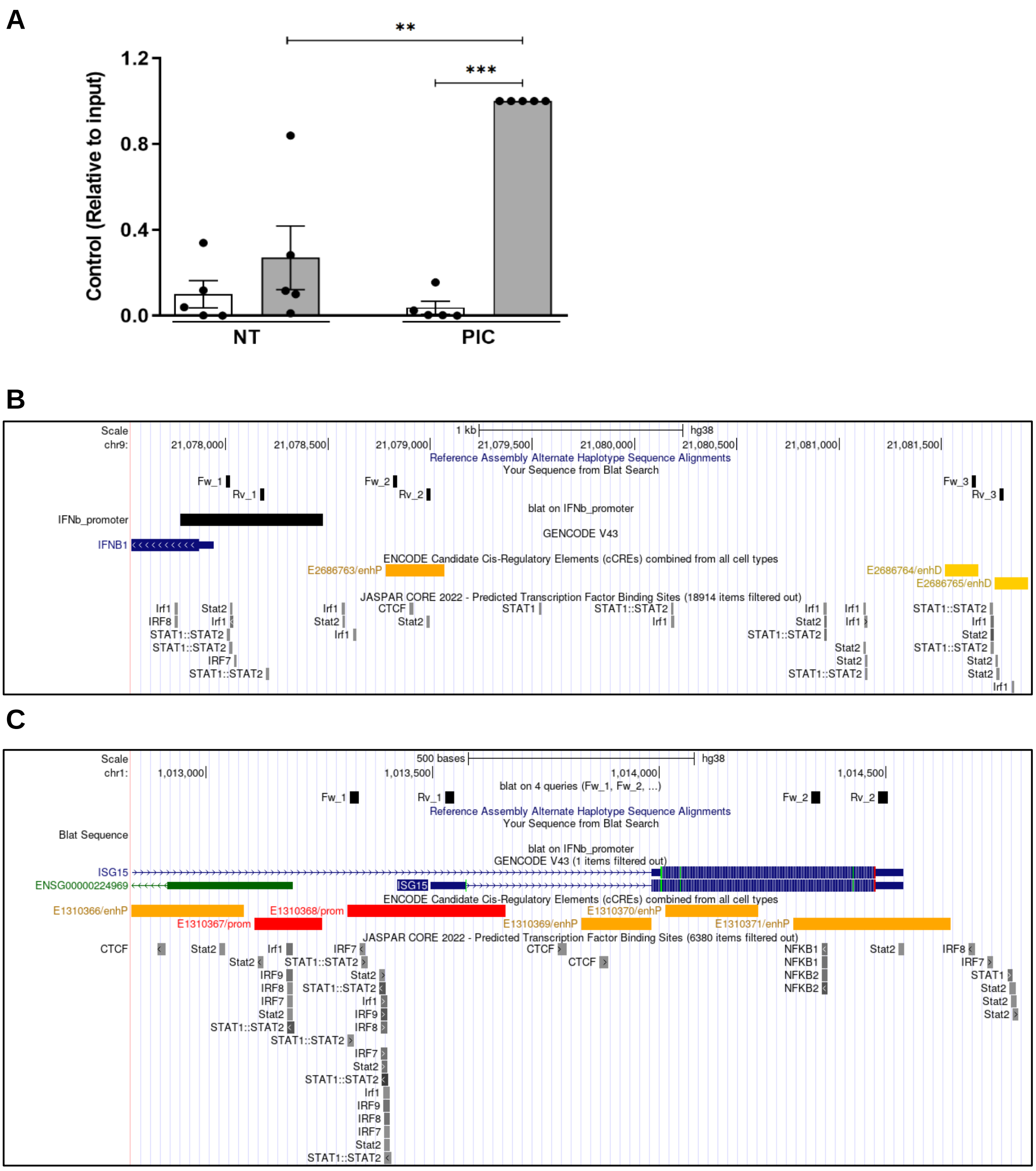

Figure S7. *ARGI* binds to the regulatory regions of *IFNβ* and *ISG15* genes upon viral insult. (A) RNA antisense purification of *ARGI* was performed in non-transfected (NT) or PIC-transfected EndoC-βH1 cells using probes targeting *ARGI* (white bars) or probes against a similar non-relevant lncRNA used as negative control (grey bars). Expression of the irrelevant control lncRNA was measured by qPCR. Results are presented as relative to input and are means±SEM of 5 independent experiments. ***$p < 0.001$ and **$p<0.01$ as indicated; ANOVA followed by Bonferroni's multiple comparisons test. (B-C) The location of the primer pairs used for the amplification of each region in the genomic map. (B) Primers FW1+RV1 were used to amplify *IFNβ* promoter, and primers FW2+RV2 and FW3+RV3 primer pairs were used to amplify two distal enhancers of *IFNβ*. (C) Primers FW1+RV1 were used to amplify ISG15 promoter and primers FW2+RV2 were used to amplify a distal enhancer of ISG15.

Table S1. List of the reagents, tools and datasets.

**Reagent/Resource**	**Reference or Source**	**Identifier or Catalog Number**
**Experimental Models**		
HEK-293	ATCC	CRL-1573™
EndoC-βH1	Human Cell Design	N/A
HEL115.6	University of Helsinki	N/A
1023A	Columbia University	N/A
Subcloning Efficiency™ DH5α Competent Cells	Invitrogen	18265017
CVB-1	ATCC	VR-28
CVB-4	ATCC	VR-184
**Recombinant DNA**		
Plasmid:*ARGI*-R	This paper	N/A
All-in-one pCas-Guide-CRISPRi vector	Origene	GE100059
pX330 vector	Addgene	42230
**Antibodies**		
Normal mouse IgG	Santa Cruz Biotechnologies	SC-2025
Rabbit polyclonal anti-CTCF	Invitrogen	PA5-17143
Anti-H3	Santa Cruz Biotechnologies	SC_1G1
Rabbit anti-human OCT4	Cell Signaling Technology	2840
Goat anti-human SOX17	R and D Systems	AF1924
Mouse anti-human NKX6.1	BD Biosciences	563022
Goat anti-human PDX1	R and D Systems	AF2419
Guinea pig anti-human insulin	Dako	A0564
Mouse anti-human glucagon	Sigma-Aldrich	G2654
Rabbit anti-human somatostatin	Abcam	AB108456
Alexa Fluor® 488 AffiniPure Donkey Anti-Mouse IgG (H+L)	Jackson ImmunoResearch Laboratories	715-545-151
Rhodamine RedTM-X (RRX) AffiniPure Donkey Anti-Goat IgG (H+L)	Jackson ImmunoResearch Laboratories	705-295-147
Alexa Fluor® 488 AffiniPure Donkey Anti-Guinea Pig IgG (H+L)	Jackson ImmunoResearch Laboratories	706-545-148
Rhodamine RedTM-X (RRX) AffiniPure Donkey Anti-Mouse IgG (H+L)	Jackson ImmunoResearch Laboratories	715-295-151
Alexa Fluor® 488 AffiniPure Donkey Anti-Rabbit IgG (H+L)	Jackson ImmunoResearch Laboratories	711-545-152
Alexa Fluor® 647 AffiniPure Donkey Anti-Rabbit IgG (H+L)	Jackson ImmunoResearch Laboratories	711-605-152
Normal mouse IgG	Santa Cruz Biotechnologies	SC-2025
Rabbit polyclonal anti-CTCF	Invitrogen	PA5-17143

Anti-H3	Santa Cruz Biotechnologies	SC_1G1
Rabbit anti-human OCT4	Cell Signaling Technology	2840
Goat anti-human SOX17	R and D Systems	AF1924
Mouse anti-human NKX6.1	BD Biosciences	563022
Goat anti-human PDX1	R and D Systems	AF2419
Guinea pig anti-human insulin	Dako	A0564
Mouse anti-human glucagon	Sigma-Aldrich	G2654
Rabbit anti-human somatostatin	Abcam	AB108456
Alexa Fluor® 488 AffiniPure Donkey Anti-Mouse IgG (H+L)	Jackson ImmunoResearch Laboratories	715-545-151
Rhodamine RedTM-X (RRX) AffiniPure Donkey Anti-Goat IgG (H+L)	Jackson ImmunoResearch Laboratories	705-295-147
Alexa Fluor® 488 AffiniPure Donkey Anti-Guinea Pig IgG (H+L)	Jackson ImmunoResearch Laboratories	706-545-148
Rhodamine RedTM-X (RRX) AffiniPure Donkey Anti-Mouse IgG (H+L)	Jackson ImmunoResearch Laboratories	715-295-151
Alexa Fluor® 488 AffiniPure Donkey Anti-Rabbit IgG (H+L)	Jackson ImmunoResearch Laboratories	711-545-152
**Oligonucleotides and other sequence-based reagents**		
ISG15_F1: TCCCTGTCTTTCGGTCATTC	This paper	N/A
ISG15_R1: ACGGCACAAGCTCCTGTACT	This paper	N/A
ISG15_F2: CACCTGAAGCAGCAAGTGAG	This paper	N/A
ISG15_R2: CTTTATTTCCGGCCCTTGAT	This paper	N/A
IFNβ_F1: TCCCACTTTCACTTCTCCCT	This paper	N/A
IFNβ_R1: GCTTTCCTTTGCTTTCTCCCA	This paper	N/A
IFNβ_F2: GGGTGGGATGGAGAACTCAG	This paper	N/A
IFNβ_R2: ACTTTTCTGTTGTTTGGTCTTGT	This paper	N/A
IFNβ_F3: GAGAACTCCTGCCCAGAGG	This paper	N/A
IFNβ_R3:AGCACCTCAAGAACACAATAGC	This paper	N/A
*ARGI*_CRISPRi sgRNA_Fw: GATCGCCGGGGATTCCCAGTTCCCC	This paper	N/A

*ARGI*_CRISPRi sgRNA_Rv: AAAACGGGAACTGGGAATCCCCGGC	This paper	N/A
*ARGI*_KO_sg1_Forward primer:CACCGCTGTAGGGACGTCTTTCCG	This paper	N/A
*ARGI*_KO_sg1_Reverse primer:AAACCGGAAAGACGTCCCTACAGC	This paper	N/A
*ARGI*_KO_sg2_Forward primer:CACCGGGATCCTTCCAAAATTGACA	This paper	N/A
*ARGI*_KO_sg2_Reverse primer:AAACTGTCAATTTTGGAAGGATCCC	This paper	N/A
*ARGI*_KO_sg3_Forward primer:CACCGGCCAGTCCCCGATCAGTGTA	This paper	N/A
*ARGI*_KO_sg3_Reverse primer:AAACTACACTGATCGGGGACTGGCC	This paper	N/A
**Chemicals, Enzymes and other reagents**		
ECM Gel from Engelbreth-Holm-Swarm murine sarcoma	Sigma-Aldrich	E1270
Fibronectin	Sigma-Aldrich	F1141
OPTIβ1®	Univercell Biosolutions	N/A
DMEM 4.5g/L Glucose w/ L-Glutamine	Lonza	H3BE12-604F
Penicillin-Streptomycin	Thermo Fisher Scientific	15140122
Polyinosinic:polycytidylic acid (PIC)	InvivoGen	31852-29-6
Invitrogen™ Lipofectamine™ 2000 Transfection Reagent	Invitrogen	10696343
Lipofectamine RNAiMAX Transfection Reagent	Invitrogen	13778150
Bay 11-7082	Sigma-Aldrich	B5556
NucleoSpin RNA, Mini kit for RNA purification	Macherey-Nagel	740955.250
PureLink™ RNA Mini Kit	Invitrogen	12183025
PrimeTime™ One-Step RT-qPCR	IDT	229298520
*ARGI*_PrimeTime qPCR Assay	IDT	Custom assay for: NONHSAT233405.1
iTaq™ Universal SYBR® Green Supermix	Bio-Rad	1725124
TruSeq® Stranded Total RNA Library Prep	Illumina	20020596
Site-Directed Mutagenesis QuickChange II	Agilent	200523

ARGI-specific (si)RNAs #1	IDT	CD.Ri.210838.13.1
ARGI-specific (si)RNAs #2	IDT	CD.Ri.210841.13.4
BamHI restriction enzyme	New England BioLabs	R0136S
BsmBI-v2 restriction enzyme	New England BioLabs	R0739S
BbsI restriction enzyme	New England BioLabs	R0539S
Dynabeads™ Protein G for Immunoprecipitation	Invitrogen	10003D
RNA Fragmentation Reagents	Invitrogen	AM8740
Streptavidin Mag Sepharose	Cytiva	28985799
NucleoSpin Gel and PCR Clean-up, Mini kit for gel extraction and PCR clean up	Machery-Nagel	740609.250
MCDB131 no Glutamine	Life Technologies	10372-019
DMEM/F12 media with GlutaMAX	Life Technologies	31331028
Essential 8™	Life Technologies	1517001
Ham’s F-10	Gibco	41550
Matrigel™ basement membrane matrix growth factor reduced, phenol red free BD	Corning	356231
IBMX	Sigma-Aldrich	15879
Accutase	A&E Scientific	ACC-1B
Accumax	Sigma-Aldrich	A7089
GlutaMAX	Life Technologies	35050
$NaHCO_3$	Merck Millipore	1.06329.0500
BSA fraction V	Sigma-Aldrich	A7030
EDTA	Life Technologies	15575020
β-mercaptoethanol	Gibco	31350-010
KSR	Life Technologies	10828010
Y-27632	Selleckchem	72304
ITS-X	Thermo Fisher Scientific	51500056
Heparin	STEMCELL Technologies	07980
Zinc sulfate	Sigma-Aldrich	Z-0251
Activin A	PreproTech	120-14E
CHIR-99021	Axon Medchem	1386
L-ascorbic acid	Sigma-Aldrich	A4554
FGF7	PreproTech	100-19
SANT-1	Sigma-Aldrich	S4572
Retinoic acid	Sigma-Aldrich	R2625
LDN-193189	Selleckchem	S2618
TPB	Santa Cruz	sc-204424
EGF	STEMCELL Technologies	78006
Nicotinamide	Sigma-Aldrich	N3376
GC-1	Tocris	4554

GSiXX	Merck Millipore	565790
ALK5 inhibitor II	ENZO	ALX-270-445-M005
Betacellulin	PreproTech	100-50
Trolox	Sigma-Aldrich	238813-1G
SP600125	Selleckchem	SP600125
Resvetratrol	Sigma-Aldrich	R5010-100mg
R428	STEMCELL Technologies	S2841
N-acetyl-cysteine	Sigma-Aldrich	A9165
Glucose solution, 2.5M stock	Sigma-Aldrich	G8769
Human total RNA master panel II	Clontech	636643
**Software**		
ggplot2package	Springer-Verlag New York	https://ggplot2.tidyverse.org/index.html
circlize package	Comprehensive R Archive Network (CRAN)	http://cran.r-project.org/web/packages/circlize/
GraphPad Prism	GraphPad Software Inc	https://www.graphpad.com/scientific-software/prism/
ImageJ	NIH	https://imagej.nih.gov/ij/
**Other**		
AggreWell™400 24 wells plate	STEMCELL Technologies	34415
AggreWell™ Rising solution	STEMCELL Technologies	07010
MX1_PrimeTime qPCR Assay	IDT	Hs.PT.58.26787898
IFIT1_PrimeTime qPCR Assay	IDT	Hs.PT.56a.2076909
IFIT3_PrimeTime qPCR Assay	IDT	Hs.PT.58.20456374
IFI6_PrimeTime qPCR Assay	IDT	Hs.PT.58.4390209
STAT1_TaqMan® Gene Expression Assay	Thermo Fisher Scientific	Hs01013996_m1
ISG15_TaqMan® Gene Expression Assay	Thermo Fisher Scientific	Hs00192713_m1
IFNβ_TaqMan® Gene Expression Assay	Thermo Fisher Scientific	Hs01077958_s1
Actinaβ_TaqMan® Gene Expression Assay	Thermo Fisher Scientific	Hs01060665_g1
MEG3_TaqMan® Gene Expression Assay	Thermo Fisher Scientific	Hs00292028_m1
RPLP0_TaqMan® Gene Expression Assay	Thermo Fisher Scientific	Hs99999902_m1
RNAseq in GEO	Gene Expression Omnibus	GSE217827
